# Development and validation of a multiplex electrochemiluminescence immunoassay to evaluate dry eye disease in rat tear fluids

**DOI:** 10.1038/s41598-023-39397-8

**Published:** 2023-07-27

**Authors:** Agnese Compagnone, An Matheeussen, Linda De Vooght, Paul Cos

**Affiliations:** Laboratory of Microbiology, Parasitology and Hygiene (LMPH), Department of Pharmaceutical Sciences, University of Antwerp, Campus Drie Eiken D.S.723, FFBD-FDFAR-LMPH, Universiteitsplein 1, 2610 Wilrijk, Antwerp Belgium

**Keywords:** Immunological techniques, Biomarkers, Diseases, Eye diseases, Immunological disorders

## Abstract

Dry eye disease (DED) is a challenge in ophthalmology. Rat models represent valuable tools to study the pathophysiology and to develop novel treatments. A major challenge in DED research is detecting multiple biomarkers in a low tear volume sample. Multiplex immunoassays for DED rat research are missing. We have developed a multiplex electrochemiluminescence immunoassay (ECLIA) to detect three biomarkers for DED: MMP-9, IL-17 and ICAM-1. Tears, used as matrix, were collected from six healthy Wistar rats. Assays were run based on the U-Plex Meso Scale Diagnostics (MSD) platform, by two independent operators according to the EMA guideline on bioanalytical method validation. Linear mixed, regression models were fit to perform the statistical analysis on the range of concentrations for the chosen analytes. During optimization, it has observed that incubation time, temperature and agitation affected the robustness of the protocol. ECLIA optimum conditions include the use of antibodies at 0.5 µg/ml concentration and 1 h incubation at room temperature with shaking. Precision met the acceptance criteria in the chosen range: 1062–133 pg/ml for ICAM-1, 275–34.4 pg/ml for IL-17, 1750–219 pg/ml for MMP-9. Accuracy and linearity were acceptable for a broader range. This is the first report of a validated ECLIA that allows measurements of three relevant DED biomarkers in rat tear fluids.

## Introduction

Dry eye disease (DED) is defined as a multifactorial disease of the tears and ocular surface that results in symptoms of discomfort, visual disturbance, and tear film instability with potential damage to the ocular surface. It is accompanied by increased osmolarity of the tear film and inflammation of the ocular surface. DED can be divided into two subtypes: aqueous-deficient and evaporative dry eye characterised by a lack of tear fluid and excessive evaporation of the tear film, respectively^1^.

Evaporative DED is characterised by Meibomian gland dysfunction (MGD). The prevalence of this pathology varies from 3.5 to 68.3% in the population, as reported in several studies^2,3^. Data on aqueous-deficient DED are more difficult to determine. Given the complexity of the disease, detailed epidemiological data are lacking, making it very difficult to evaluate the actual prevalence, characteristics, development, and outcomes of this disease^4^. Research into the biomarker diagnostic field is required to ease the process of fitting the disease into defined parameters^5^.

To elucidate and to evaluate novel potential treatments for DED, a variety of different animal species were employed to create animal models for DED, including mice, rats, rabbits, dogs, and primates. Even though findings in these animal models cannot literally be extrapolated to humans, it does provide a valuable tool to obtain a first proof-of-concept. Using rodents like mice and rats has several advantages, such as easier handling, reduced costs, fewer prerequisites for housing and maintenance^6^. Greater ocular dimension and tear volume made rats superior in this type of research, in comparison to mice. Two major disadvantages of using rats are the low tear volume sample and the lack of existing rat-based multiplex immunoassays for three major biomarkers of interest, i.e. matrix metallo-proteinase 9 (MMP-9), interleukin 17 (IL-17) and intercellular adhesion molecule 1 (ICAM-1).

Matrix metallo-proteinases (MMPs) have been shown to alter the corneal epithelium barrier and disease or dysfunction of the lacrimal functional unit alters the balance of MMPs^7^.

The level or activity of MMP-9 in tear samples has been evaluated frequently and it has been shown that elevated values of this biomarker have a strong and positive correlation and specificity with clinical diagnosis of DED^8–10^. In DED, triggering events induce the activation of the Th17 cells pathway, producing the pro-inflammatory IL-17^11–13^. Its role as a marker for DED is being mentioned by abundant literature^14,15^. ICAM-1 is involved in signaling transmigration of lymphocytes to inflammatory sites and together with its receptor, the lymphocyte functional associated antigen-1 (LFA-1), it induces the T-cell activation^16,17^. FDA approved in 2016 the drug Lifitegrast that limits the interaction between ICAM-1 and LFA-1 and reduces the inflammation in DED patients^18^. ICAM-1 has been used as an indicator of DED in several clinical and pre-clinical studies^15,19–22^.

Dry eye disease (DED) is still a disease with many blind spots, starting from the etiology and the pathophysiology to the treatments that are unable to break the vicious cycle of DED, making it more chronic and severe. Animal models have contributed significantly to our current understanding of DED pathogenesis and are necessary to further unravel DED pathophysiology and to evaluate novel therapies for human as well as veterinary use^23–27^.

The animal models can mirror several mechanisms responsible for DED, such as insufficient lacrimal gland production, Meibomian gland dysfunction and environmental stress. Therefore, it is pivotal to develop and validate methods specific for animal models of DED that can assess parameters relevant for the disease^24,28,29^.

The novelty of this study lies in its first ready-to-use rat-based multiplex immunoassay that allows quantification of three major biomarkers of DED specifically in rat tear fluids. In contrast to the use of ocular tissues, tear fluids have the advantage to be collected in a non-invasive way and at different time points. Given the chronic character of DED, a longitudinal study of the disease is essential and our assay allows this. Moreover, the three analytes involved in the validation are not only related to DED, but are also involved in other ocular diseases, making the assay available for a broader rat model-based scientific community^30–32^.

The rationale behind this multiplex ECLIA on rat tear fluids is represented by the use of rat animal models in DED research and the need of analysing the progress of the disease in the animal model. With DED animal models there is often a very limited amount of sample, e.g. tear fluids. Moreover, there is a lack of commercially available assays for detecting these three crucial biomarkers in a single assay. This led to the development and the validation of this novel method for rat-based research. Instead, these needs are well covered in human DED research.

Therefore, according to the EMA guideline on bioanalytical method validation^33^, we have developed and validated a multiplex ECLIA to detect three relevant biomarkers for DED, i.e. MMP-9, IL-17 and ICAM-1, in rat tear fluid.

## Results

Before the validation with rat tear fluids, a preliminary study was performed on the assay diluent as matrix. The whole process and the data are listed in the Supplementary files.

### ECLIA with tear fluid matrix

**Figure 1:**
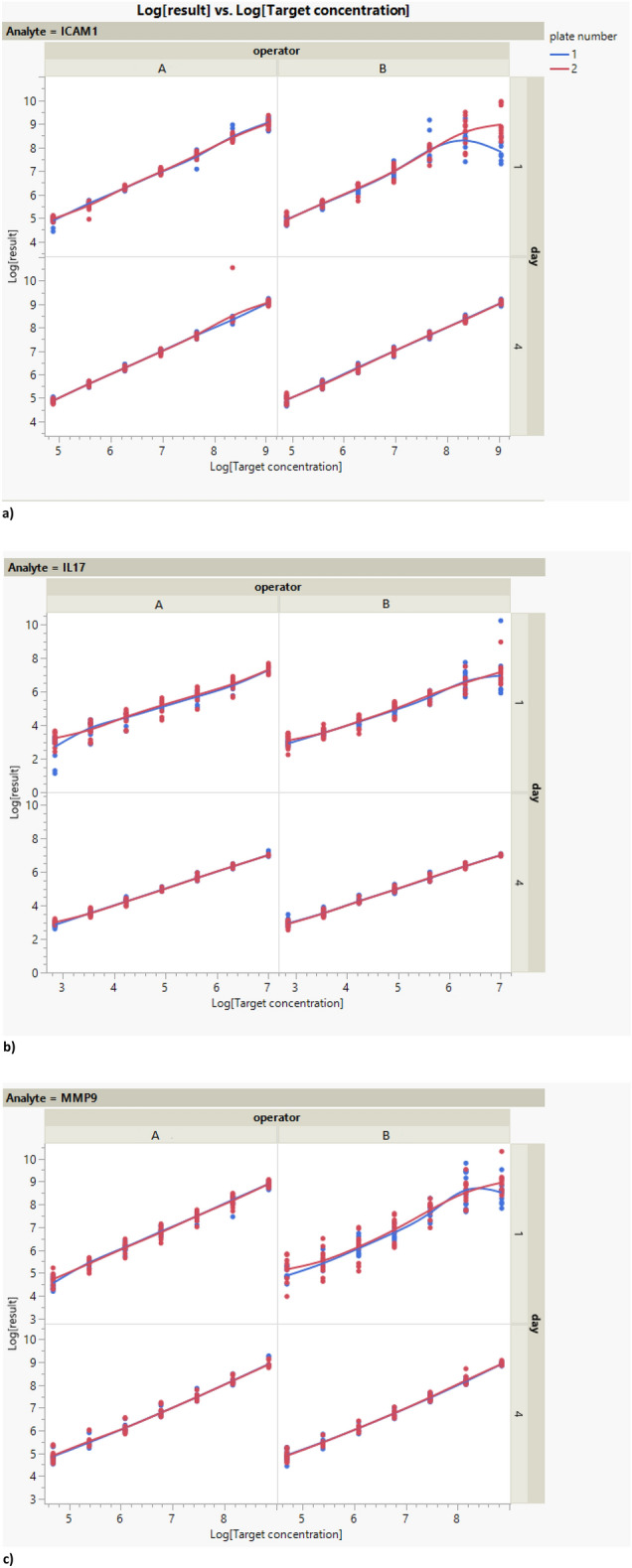
Graph of the data after logarithmic transformation (natural base) versus target concentration after logarithmic transformation (natural base) per analyte, operator, day and plate. N = 6 samples run in duplicates.

Using collected tear fluid as matrix several parameters were evaluated. Two identical assays in two different days were run by two operators with two technical repetitions for 6 animals. Data depict the relationship between the log transformed results versus the log transformed target concentration for the three analytes: ICAM-1, IL1-7 and MMP-9 for the two operators during the 2 days. There is visually a relatively linear relationship between the log results and the log target concentration with a small alteration at higher concentrations for the three analytes when analysed by operator B at day 1, as shown in Fig. 1. All standard curves from the assays are shown in the Supplementary file 4.

### Repeatability and intermediate precision

The variance components were estimated for each analyte and for each concentration level. Table 1 shows the CV (%) of the day to day, operator to operator and plate to plate effects as well as the pure repeatability variability (under same operating conditions: intra-assay precision) and the overall one, also called intermediate precision variability (within laboratory variations, e.g. different days) of the assay. In addition, the upper 95% confidence limit of the repeatability CVs and intermediate precision CVs are reported.

**Table 1:** Precision: CVs per analyte per concentration level together with their corresponding upper 95% confidence limits.

Analyte	Target concentration	Day CV	Operator CV	Plate CV	Repeatability CV	Intermediate precision CV	95% upper confidence limit of CV repeatability	95% upper confidence limit of CV intermediate precision
ICAM-1	133	0.00	0.00	0.00	14.52	14.52	16.96	16.96
ICAM-1	266	0.00	0.00	0.00	12.39	12.39	14.46	14.46
ICAM-1	531	0.00	0.00	0.00	13.44	13.44	15.69	15.69
ICAM-1	1062	0.00	0.00	0.00	16.19	16.19	18.91	18.91
ICAM-1	2125	8.62	4.85	0.00	24.20	26.26	28.44	33.31
ICAM-1	4250	0.00	0.00	4.00	38.37	38.61	45.98	45.93
ICAM-1	8500	17.02	0.00	42.06	28.01	55.32	33.28	105.95
IL-17	17.2	0.00	0.00	12.14	37.64	39.82	44.78	47.72
IL-17	34.4	11.35	2.55	0.00	28.19	30.67	33.18	38.70
IL-17	68.8	10.40	0.00	0.00	27.31	29.36	32.13	36.14
IL-17	138	8.54	0.00	0.00	27.67	29.05	32.56	34.98
IL-17	275	1.90	0.00	0.00	27.65	27.72	32.53	32.54
IL-17	550	9.09	0.00	0.00	36.13	37.40	42.71	44.75
IL-17	1100	10.21	0.00	0.00	50.75	52.03	60.76	62.66
MMP-9	109	10.70	11.70	7.61	31.68	36.52	37.50	51.50
MMP-9	219	0.00	0.00	0.00	27.63	27.63	32.42	32.42
MMP-9	438	0.00	0.00	0.00	29.22	29.22	34.32	34.32
MMP-9	875	0.00	0.00	0.00	26.60	26.60	31.20	31.20
MMP-9	1750	8.34	8.34	0.00	24.50	25.96	28.78	31.45
MMP-9	3500	20.31	20.31	0.00	36.71	42.68	43.46	59.48
MMP-9	7000	0.00	0.00	12.62	26.77	29.79	31.75	36.65

Target concentrations are expressed in pg/ml. CV values are expressed in %. N = 6 samples run in duplicates.

For ICAM-1, most of the assay variability is related to the pure repeatability for concentration levels smaller than 1062 with an Intermediate precision CV lower than 20%. For higher concentration levels, plate and day sources of variability increase and the intermediate precision CV rises from 26% up to 55%.

For IL-17, most of the variability components influence intermediate precision CV whatever the concentration level. These intermediate precision CVs range between 28 and 40% for all levels except the highest concentration level for which the CV rises up to 52%.

For MMP-9, some of the variability components influence the intermediate precision CV but not for all the concentration levels. The intermediate precisions CVs range between 28 and 43% for all concentration levels.

**Figure 2:**
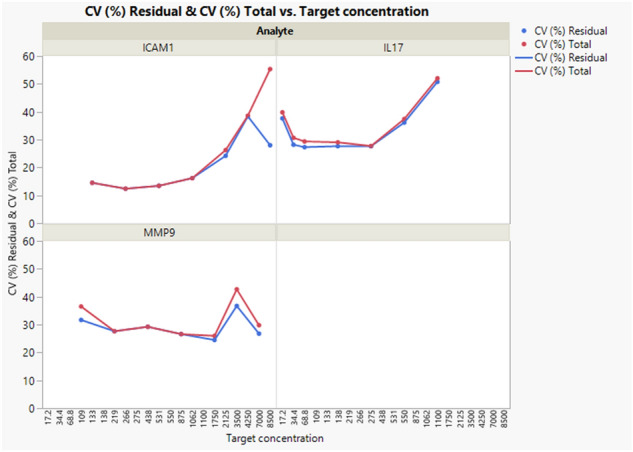
Precision—evolution of the repeatability (residual) and intermediate precision (total) CVs per analyte per concentration level. Target concentrations are expressed in pg/ml. N = 6 samples run in duplicates.

Figure 2 represents the evolution of the repeatability and intermediate precisions CV across the target concentrations.

Table 2 gives the CV of the analytical method for each analyte whatever the concentration level of the analyte. As can be seen, the intermediate precisions are 26%, 36% and 31% for ICAM-1, IL-17, MMP-9, respectively.

**Table 2:** Precision: CVs per analyte for overall concentration levels together with the corresponding upper 95% confidence limits for repeatability and intermediate precision CV.

Analyte	Variability source	CV	95% upper confidence limit of CV
ICAM-1	Operator	0.00	
ICAM-1	Day	0.00	
ICAM-1	Plate	3.61	
ICAM-1	Repeatability	26.10	27.67
ICAM-1	Intermediate precision	26.37	27.97
IL-17	Operator	0.00	
IL-17	Day	7.50	
IL-17	Plate	2.84	
IL-17	Repeatability	34.65	36.76
IL-17	Intermediate precision	35.67	38.44
MMP-9	Operator	2.68	
MMP-9	Day	3.75	
MMP-9	Plate	3.66	
MMP-9	Repeatability	30.49	32.32
MMP-9	Intermediate precision	31.10	33.27

N = 6 samples run in duplicates.

### Trueness

**Figure 3:**
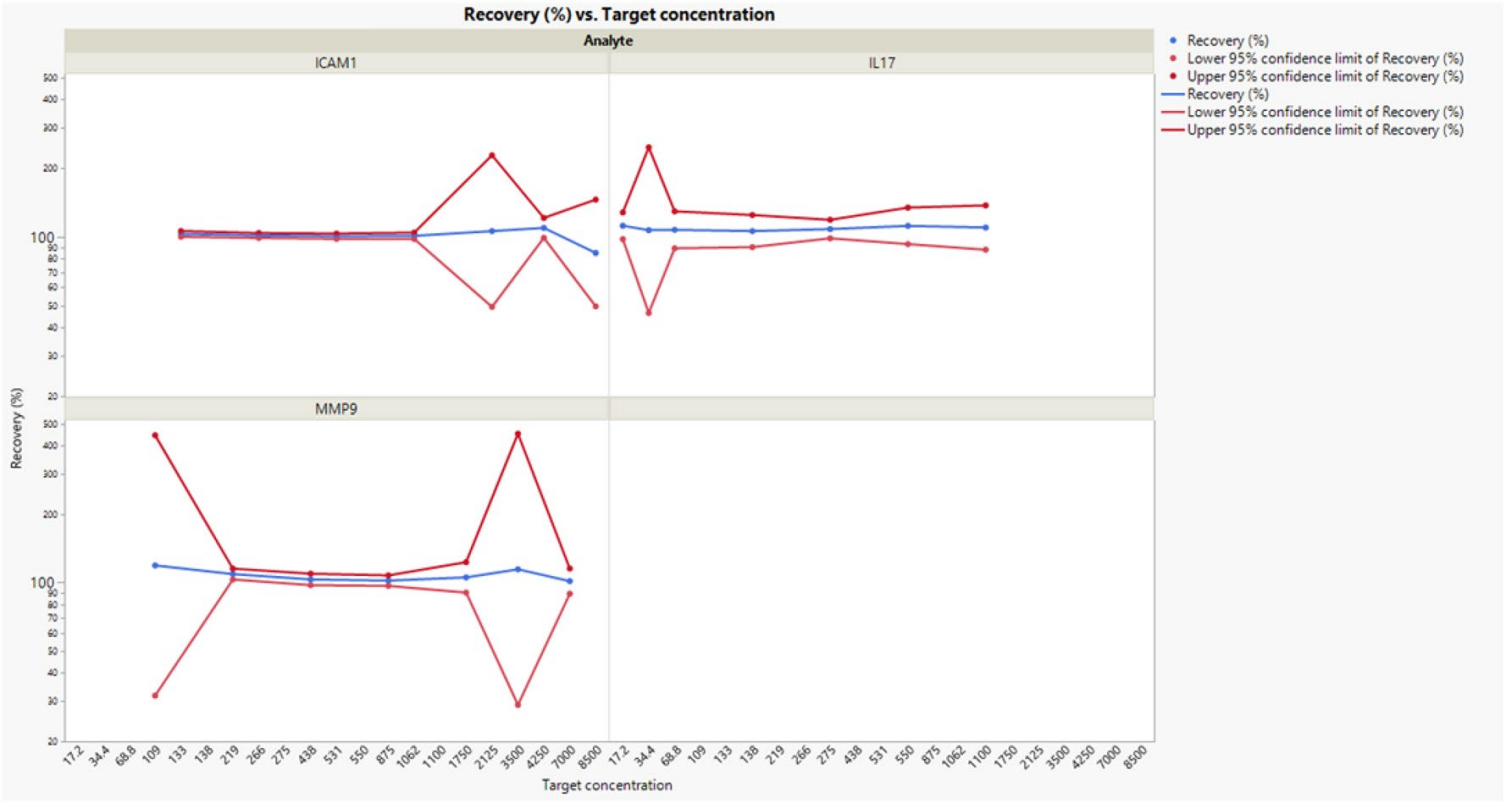
Trueness: evolution of recoveries (%) across concentration levels per analyte together with their corresponding 95% confidence intervals. Target concentrations are expressed in pg/ml. N = 6 samples run in duplicates.

Table 3 and Fig. 3 show the trueness of the analytical method per analyte and per concentration level, expressed in terms of recoveries (%) with their 95% confidence intervals. For all analytes the recoveries are greater than 100%, showing an overestimation of the target concentration, except for ICAM1 at concentration level 8500.

**Table 3:** Trueness: recoveries (%) per analyte and concentration levels together with their corresponding 95% confidence intervals.

Analyte	Target concentration (pg/ml)	Recovery (%)	Lower 95% confidence limit of recovery (%)	Upper 95% confidence limit of recovery (%)
ICAM-1	133	102.89	99.92	105.95
ICAM-1	266	101.23	98.73	103.80
ICAM-1	531	100.52	97.83	103.28
ICAM-1	1062	100.92	97.68	104.26
ICAM-1	2125	105.84	49.27	227.34
ICAM-1	4250	109.33	98.89	120.89
ICAM-1	8500	84.89	49.55	145.43
IL-17	17.2	111.68	97.60	127.80
IL-17	34.4	106.85	46.36	246.27
IL-17	68.8	107.21	88.96	129.20
IL-17	138	105.84	90.02	124.43
IL-17	275	108.01	98.40	118.56
IL-17	550	111.57	92.83	134.08
IL-17	1100	109.68	87.72	137.14
MMP-9	109	118.76	31.79	443.68
MMP-9	219	108.87	103.05	115.02
MMP-9	438	103.10	97.29	109.26
MMP-9	875	101.85	96.59	107.39
MMP-9	1750	105.23	90.22	122.75
MMP-9	3500	114.23	28.95	450.80
MMP-9	7000	101.33	89.22	115.07

N = 6 samples run in duplicates.

For ICAM-1 recoveries range from 85 to 109%, for IL-17 from 106 to 112% and for MMP-9 from 101 to 119%. The graph shows that spiked observations in the utmost recovery confidence limits mirror the high intermediate variance evident in precision data.

Considering IL-17, confidence intervals are wider as a consequence of the further sources of variability.

### Linearity

**Figure 4:**
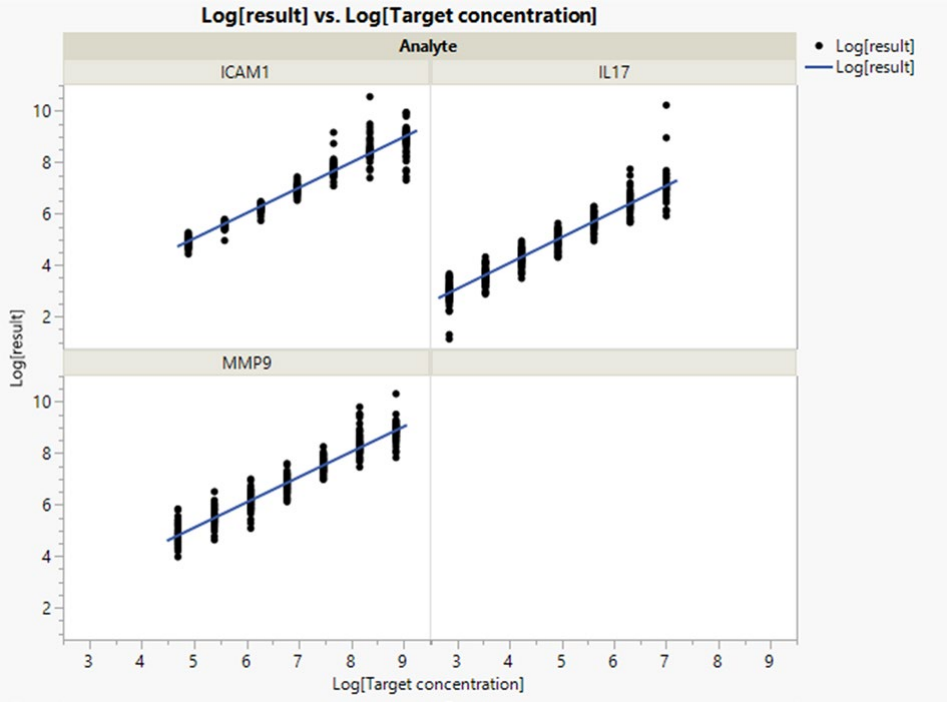
Linearity: regression graph and raw data per analyte. N = 6 samples run in duplicates.

Figure 4 illustrates the data fitting a linear regression except for the two highest concentrations. For that reason, the R^2^ (determination coefficient) values are below 97%. Table 4 gives the (R^2^) of the linear models for each analyte and the slopes and intercepts of the linear model fitted for each analyte with their respective 95% confidence intervals. The slopes are close to 1 and the intercepts close to 0, suggesting that there is a significant linear relationship between the results and target concentration.

**Table 4:** Linearity: slope and intercept per analyte, with their corresponding 95% confidence intervals and determination coefficient (R^2^) per analyte.

Analyte	Parameter	Estimate	Lower 95% confidence limit	Upper 95% confidence limit	R^2^
ICAM-1	Intercept	0.10	− 0.0084	0.1987	0.9639
ICAM-1	Slope	0.99	0.9734	1.0026	
IL-17	Intercept	0.07	− 0.0224	0.1704	0.9423
IL-17	Slope	1.00	0.9830	1.0207	
MMP-9	Intercept	0.19	0.0758	0.3074	0.952
MMP-9	Slope	0.98	0.9657	0.9992	

N = 6 samples run in duplicates.

### Robustness

**Figure 5:**
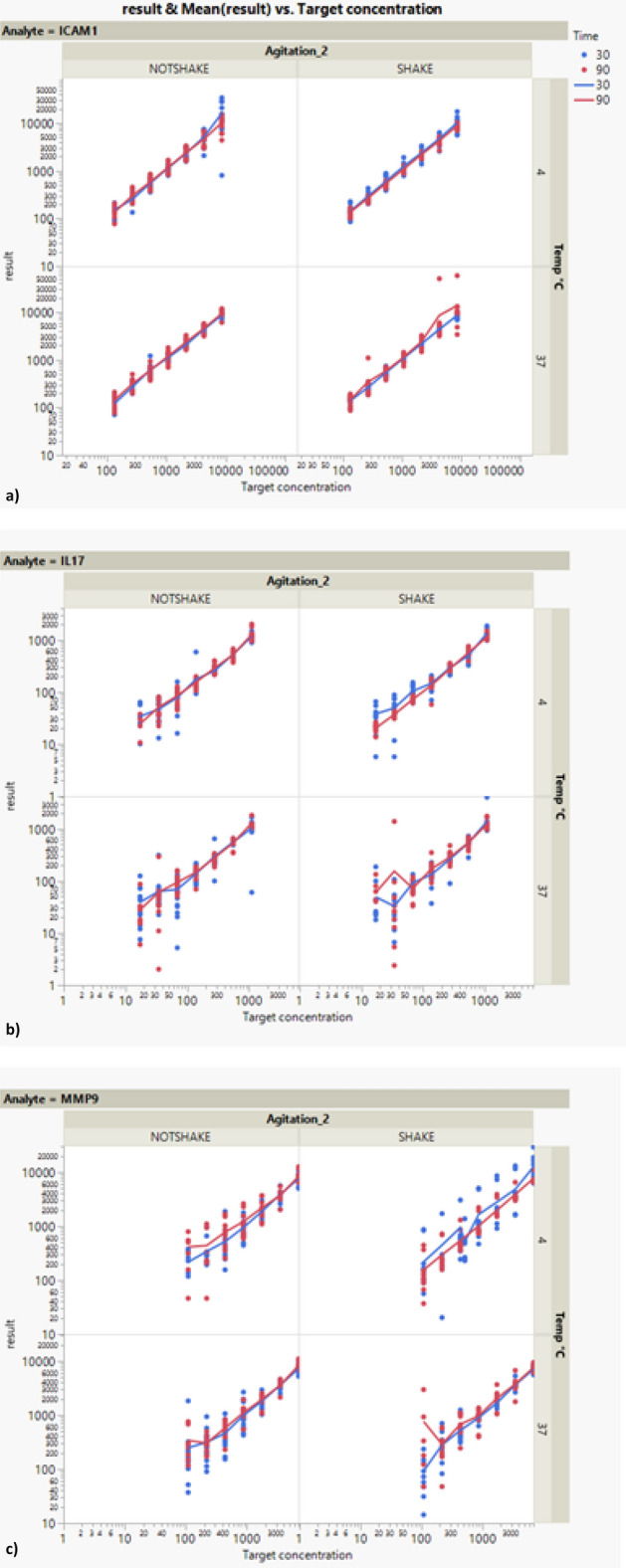
Results of the robustness experiments when changing the time between 30 and 90 min, the temperature between 4 and 37 °C and with or without agitation for the three analytes: ICAM-1, IL-17 and MMP-9. N = 6 samples run in duplicates.

Figure 5 shows the results of the robustness experiments when changing the “time of incubation” parameter (30 and 90 min), the “temperature of incubation” parameter (4 °C and 37 °C) and with or without agitation (SHAKE/NOTSHAKE) for the three analytes.

**Figure 6:**
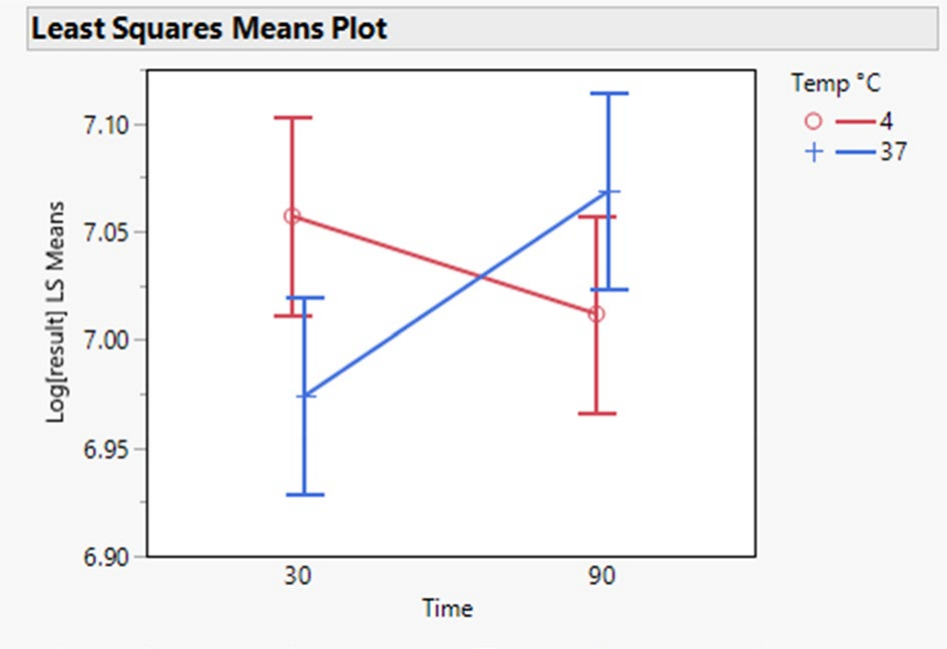
ICAM-1—least squares means of the effect of temperature and time on the log transformed results of ICAM-1 showing the interaction between these two factors. Time values are expressed in minutes. N = 6 samples run in duplicates.

Table 5 shows the statistical significance of the parameters studied for the three analytes. The last column of this table gives the p value (Prob > F). There is a statistically considerable effect of the target concentration on the results measured for ICAM-1. Therefore, there is not a random relationship between the target concentration and measured values. There is also a statistically significant effect of time and temperature interaction (p value = 0.0026). This means that the effect of time on the results of ICAM-1 depends on the value of the temperature. This is illustrated in Fig. 6 showing that when time increases, there is an increase in the results of ICAM-1 at 37 °C, whereas there is a small decrease at 4 °C.

**Table 5:** ICAM1, IL-17 and MMP-9—statistical significance of the parameters of the model fitted to the robustness data.

Source	Nparm	DF	Sum of squares	F ratio	Prob > F
Effect tests ICAM-1					
Time	1	1	0.1027	1.1351	0.2871
Temp °C	1	1	0.0288	0.3183	0.5728
Agitation_2	1	1	0.000263	0.0029	0.9571
Time * Temp °C	1	1	0.8244	9.1094	0.0026*
Temp °C * Agitation_2	1	1	0.1774	1.9597	0.162
Time * Agitation_2	1	1	0.000138	0.0015	0.9689
Log[Target concentration]	1	1	1343.098	14,840.86	< 0.0001*
Log[Target concentration] * Time	1	1	0.0215	0.2375	0.6262
Log[Target concentration] * Temp °C	1	1	0.318	0.3518	0.5533
Log[Target concentration] * Agitation_2	1	1	0.0352	0.3888	0.5332
Effect tests IL-17					
Time	1	1	0.0131	0.0425	0.8367
Temp °C	1	1	0.3214	1.0406	0.3081
Agitation_2	1	1	0.0422	0.1368	0.7116
Time * Temp °C	1	1	0.2189	0.7089	0.4001
Temp °C * Agitation_2	1	1	0.0403	0.1304	0.7181
Time * Agitation_2	1	1	0.3539	1.1458	0.2848
Log[Target concentration]	1	1	1033.848	334.,396	> 0.0001*
Log[Target concentration] * Time	1	1	1.2755	4.13	0.0425*
Log[Target concentration] * Temp °C	1	1	0.000103	0.0003	0.9854
Log[Target concentration] * Agitation_2	1	1	0.556	1.8001	0.1802
Effect tests MMP-9					
Time	1	1	2.2604	6.9407	0.0086*
Temp °C	1	1	0.4011	1.2315	0.2675
Agitation_2	1	1	0.1496	0.4592	0.4982
Time * Temp °C	1	1	0.9092	2.7917	0.0952
Temp °C * Agitation_2	1	1	0.4541	1.3942	0.2381
Time * Agitation_2	1	1	1.6116	4.9484	0.0264*
Log[Target concentration]	1	1	1053.979	3236.337	< 0.0001*
Log[Target concentration] * Time	1	1	2.9089	8.932	0.0029*
Log[Target concentration] * Temp °C	1	1	0.3978	1.2214	0.2695
Log[Target concentration] * Agitation_2	1	1	1.6413	5.0396	0.0251*

The column Prob > F shows the *p *value of the corresponding effect. “Nparm” and “DF” columns indicate respectively the number of parameters and degrees of freedom of the analysis. N = 6 samples run in duplicates.

Table 6 shows the difference in log scale of all combinations of time and temperature with their 95% confidence intervals. Further, it shows same results but expressed as recoveries. The only statistically considerable effect is between the condition of [90 min, 37] and [30 min, 37 °C], with a mean recovery of 109.94%. Thus, working at [90 min, 37 °C] increases the results of ICAM1 up to approximately 110% by comparison to working at [30 min, 37 °C]. Although not statistically significant, there is also a relatively strong effect between working at [30 min, 4 °C] and at [30 min, 37 °C], with a recovery of 108.67%.

**Table 6:** ICAM-1—differences in log scale of all combinations of time (minutes) and temperature (°C) together with their 95% confidence intervals.

Level (time, temperature)	Minus level (time, temperature)	Difference in Log	Lower 95% CL of difference in Log	Upper 95% CL of difference in Log	Recovery (%)	Lower 95% CL of recovery (%)	Upper 95% CL of recovery (%)	p Value
90, 37	30, 37	0.0948	0.0102	0.1793	109.94	101.03	119.64	0.0209
30, 4	30, 37	0.0831	− 0.0014	0.1677	108.67	99.86	118.26	0.0559
90, 37	90, 4	0.0570	− 0.0276	0.1415	105.86	97.28	115.20	0.3062
30, 4	90, 4	0.0453	− 0.0392	0.1299	104.64	96.15	113.87	0.5118
90, 4	30, 37	0.0378	− 0.0467	0.1224	103.85	95.44	113.02	0.6572
90, 37	30, 4	0.0116	− 0.0729	0.0962	101.17	92.97	110.10	0.9847

The corresponding recoveries are also given together with their 95% confidence intervals. The last column shows the p value of the differences tested. N = 6 samples run in duplicates.

Table 5 shows the statistical significance of the parameters studied for the analyte IL-17.

There is a substantial effect of the target concentration on the results measured for IL-17. Moreover, there is a statistically significant effect of the time and target concentration interaction (p value = 0.0425). The slope of the target concentration depends on the time. With 30 min of incubation the slope of the target concentration decreases, whereas with 90 min of incubation it increases.

The statistical significance of the parameters for MMP-9 can be observed in Table 5.

**Figure 7:**
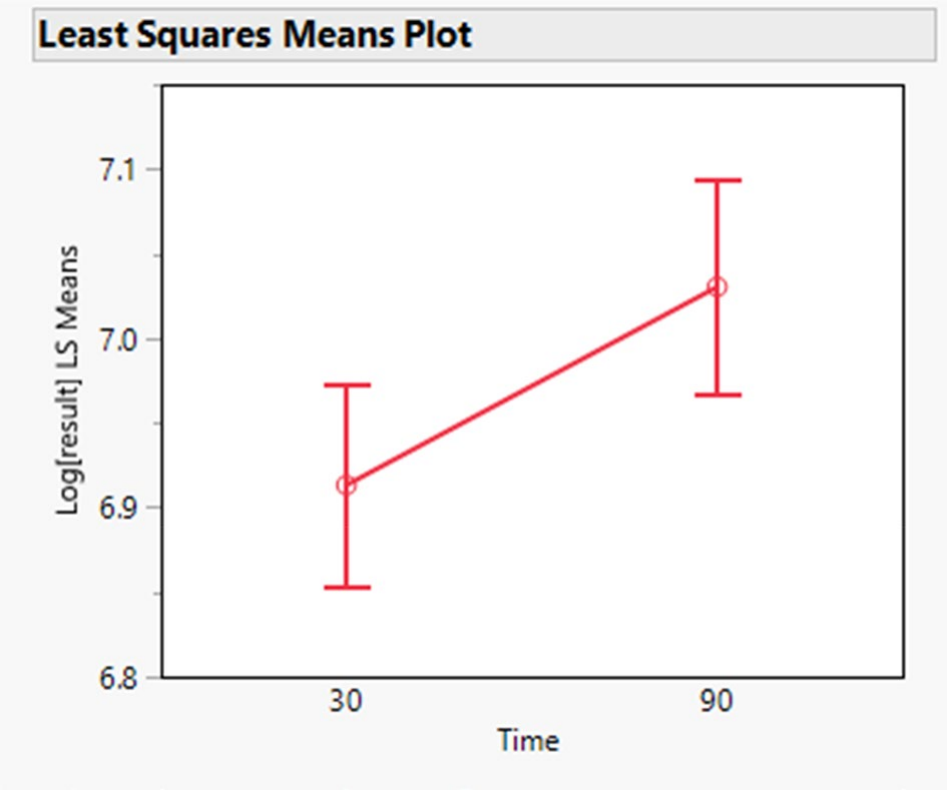
MMP-9—least squares means of the effect of time (minutes) on the log transformed results of MMP-9 showing the time factor N = 6 samples run in duplicates.

There is a statistically significant effect of the target concentration on the MMP9 results (p value < 0.0001). In addition, a statistically significant effect is visible around time and target concentration interaction (p value = 0.0029). The slope of the target concentration depends on the time. With 30 min of incubation the slope of target concentration increases, whereas with 90 min it decreases. Moreover, there is a statistically significant effect around the agitation and target concentration interaction (p value = 0.0251). Without agitation the slope of target concentration decreases, whereas with agitation it increases. Another statistically significant effect is present around time of incubation (p value = 0.0086), which is illustrated in Fig. 7. When time increases, there is an increase in the results of MMP-9.

Table 7 shows the difference in log scale with their 95% confidence interval as well as expressed as recoveries: going from 30 to 90 min of incubation increases the results of MMP-9 on average by 112.43%.

**Table 7:** MMP-9—difference in log scale of time 90 min and time 30 min together with the 95% confidence interval.

Level (time, temperature)	Minus Level (time, temperature)	Difference in Log	Lower 95% CL of difference in Log	Upper 95% CL of difference in Log	Recovery (%)	Lower 95% CL of recovery (%)	Upper 95% CL of recovery (%)	p value
90	30	0.1172	0.0298	0.2045	112.43	103.03	122.69	0.0086
90, NOT SHAKE	30, NOT SHAKE	0.2161	0.056	0.3762	124.12	105.76	145.68	0.003
90, NOT SHAKE	30, SHAKE	0.1473	− 0.0189	0.3135	115.87	98.12	136.83	0.1032
90, NOT SHAKE	90, SHAKE	0.129	− 0.038	0.2961	113.77	96.27	134.46	0.1929
90, SHAKE	30, NOT SHAKE	0.0871	− 0.0705	0.2446	109.1	93.19	127.71	0.4852
30, SHAKE	30, NOT SHAKE	0.0688	− 0.0879	0.2255	107.12	91.59	125.29	0.6705
90, SHAKE	30, SHAKE	0.0183	− 0.1456	0.1821	101.84	86.45	119.97	0.9918

Differences in log scale of all combinations of time and agitation. The corresponding recovery is also given together with its 95% confidence intervals. The last column shows the *p *value of the difference tested. N = 6 samples run in duplicates.

**Figure 8:**
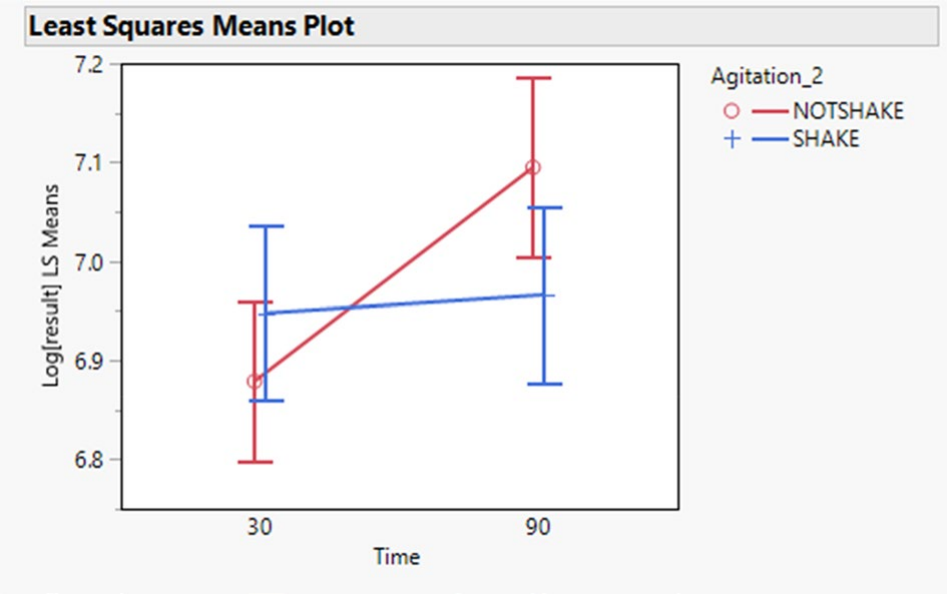
MMP-9—least squares means of the effect of time (minutes) and agitation on the log transformed results of MMP9 showing the interaction between these two factors. N = 6 samples run in duplicates.

An important statistically significant effect of time and agitation interaction is shown in Table 5 (p value = 0.0264). The effect of time on the results of MMP-9 depends on the level of the agitation. Figure 8 shows that when time increases, there is an increase in the results of MMP-9 without agitation (NOTSHAKE), whereas there is almost no effect with agitation (SHAKE).

Table 7 conveys the difference in log scale of all combinations of time and agitation together with their 95% confidence intervals. Same results are also expressed as recoveries.

The most statistically substantial impact is between the condition of [90 min, NOT SHAKE] and [30 min, NOT SHAKE], with a mean recovery of 124.12%, therefore working at [90 min, NOT SHAKE] increases the results of MMP-9 by approximately 124% by comparison to [30 min, NOT SHAKE].

## Discussion

To minimise the use of tear fluids from rats, preliminary data were obtained using the spiked assay diluent as matrix. Results from these preliminary assays fit the requirements for the validation, being dilution linearity, precision, trueness, and robustness.

After optimization with the matrix, validation of the multiplex ECLIA was performed on rat tear fluids. It is important to note that the assays run with the assay buffer matrix were performed by one single operator with previous experience in immunoassays, limiting the variability in the results. Moreover, the upper and lower limit of quantification (ULOQ and LLOQ) correspond to the utmost values of the chosen dynamic range.

The experiments with tear fluid matrix was performed by two operators which certainly increased the variability. The assays for testing robustness were performed by one operator only, considering that inter and intra-assay parameters were not evaluated in the robustness experimental design. When evaluating precision, several variations are located in the highest and lowest concentrations and, especially the IL-17 analyte was influenced by the “day” variable. It is pivotal to define a detection range, ULOQ and LLOQ. From the statistical analysis performed, the detection range could include all the concentrations ≤ 30 CV: ICAM-1 starting from 1062 to 133 pg/ml, IL-17 from 275 to 34.4 pg/ml and for MMP-9 from 1750 to 219 pg/ml. With regard of these dynamic ranges, the lowest and the highest concentrations respectively represent the LLOQ and ULOQ.

Intra-assay precision (repeatability) is a measure of the variance between data points within an assay and inter-assay precision (intermediate precision) is a measure of the variance between runs of sample replicates on different plates.

Repeatability and intermediate precision CV values were considered in the evaluation of the detection range. It is pivotal to mention that in biological assays it is possible to have large inter-assay variation that can be caused by intrinsic biological variation. This contingency has to be considered when determining acceptance criteria for assay efficiency. In this case, precision has been established at maximum 30% CV.

According to EMA guidelines for analytical methods validation, between-run and within-run precision CV values for LLOQ should not exceed 20%.

The CV value cap applied allows to use the assay, nevertheless considering the further optimisation required and the absence of a commercially available multiplex immunoassays able to detect these three analytes.

From the precision data it is evident that confidence intervals are spiked, because of the reduced number of operators (n = 2) and eventual variations in the results make confidence intervals reasonably wide. Indeed, CV values of intermediate precision are higher which suggests that are influenced by “day”, “operator” and “plate” factors. This implies that inexperience and avoidable errors could affect results. Unfortunately, the cost of assays also affects the repetitions of eventual assays.

A valid range is where linearity, recovery and precision are satisfactory. Accuracy range is within the parameters for all values, since an acceptable %Recovery varies from 80 to 120^34^. Additionally, linearity is satisfied, since the slopes close to 1 and the intercepts close to 0 suggest that there is an acceptable linear relationship between the result and the target concentration.

Considering the robustness analysis, conditions in which alteration of results is visible are time, temperature and agitation, especially for the MMP-9 analyte.

Nevertheless, the ECLIA can be used in the dynamic ranges indicated and, eventually, when the CV for the respective concentration value is not satisfied, the correspondent correction factor (1) could be used, which is the inverse of %Recovery:

1 $$value\frac{\pm 2CV\left(\%\right)x}{100}$$

where *value* is the target concentration of that specific CV and x is the estimation obtained from the immunoassay.

The experiments and analysis show that this assay is well developed and very promising for a definitive validation, according to the EMA guidelines. The indicators of precision tend to be slightly over the range of acceptance reported by EMA^33^, but this should encourage the optimization of the assay and the improvement of the precision parameter of the analytes, considering the expertise of operators and the availability of repetitions.

In the meanwhile though, the assay can be used and the correction factor can be appropriately adopted, when necessary, and the determined dynamic ranges can be employed.

Concerning optimization, it should also be important to start including more operators and more days for harmonizing results and having better understanding of the errors in the assay.

In comparison to ELISAs for the three biomarkers, our multiplex ECLIA shows less precise values, but is comparable in terms of accuracy. The detection range is often a challenge in immunoassays, but this ECLIA presents a comparable or even broader detection range than the available kits. The ECLIA technique is gaining popularity as it appears to be more stable, robust, with significant linearity and it requires less amount of sample. They also perform better when it comes to multiplexing^35,36^. Several studies showed that ECLIAs outperform standard analytical techniques like ELISA^37–40^.

## Methods

### Samples

Tear fluids matrix was collected from six Female Wistar rats (200–300 g, Janvier Labs, Roubaix, France) who were kept under standard pathogen-free conditions. Husbandry conditions were room temperature 20–25 °C, humidity 50–60% and a day–night cycle of 12 h light/12 h dark. Food and water were available ad libitum^41^. Wistar rats were managed in accordance with the guidelines provided by the European Directive for Laboratory Animal Care (Directive 2010/63/EU of the European Parliament). The laboratory Animal Ethics Committee of the University of Antwerp authorised and approved all animal experiments in this study (approval number 2019-07). All research followed the ARRIVE (Animal Research: Reporting of In Vivo Experiments) guidelines.

Tear fluid was collected twice a week for 1 month from both eyes with 10 µl capillaries (Blaubrand Intramark, Wertheim, Germany) connected with a flexible tube to a syringe located in the medial cantus of the eye. Prior to tear fluid collection, animals were sedated with 2% isoflurane (ISOFLO^®^, Zoetis, UK) until completion of tear fluid collection. Right after, capillaries and tear fluids were stored at − 80 °C^41^.

### Assay procedure and equipment

**Figure 9:**
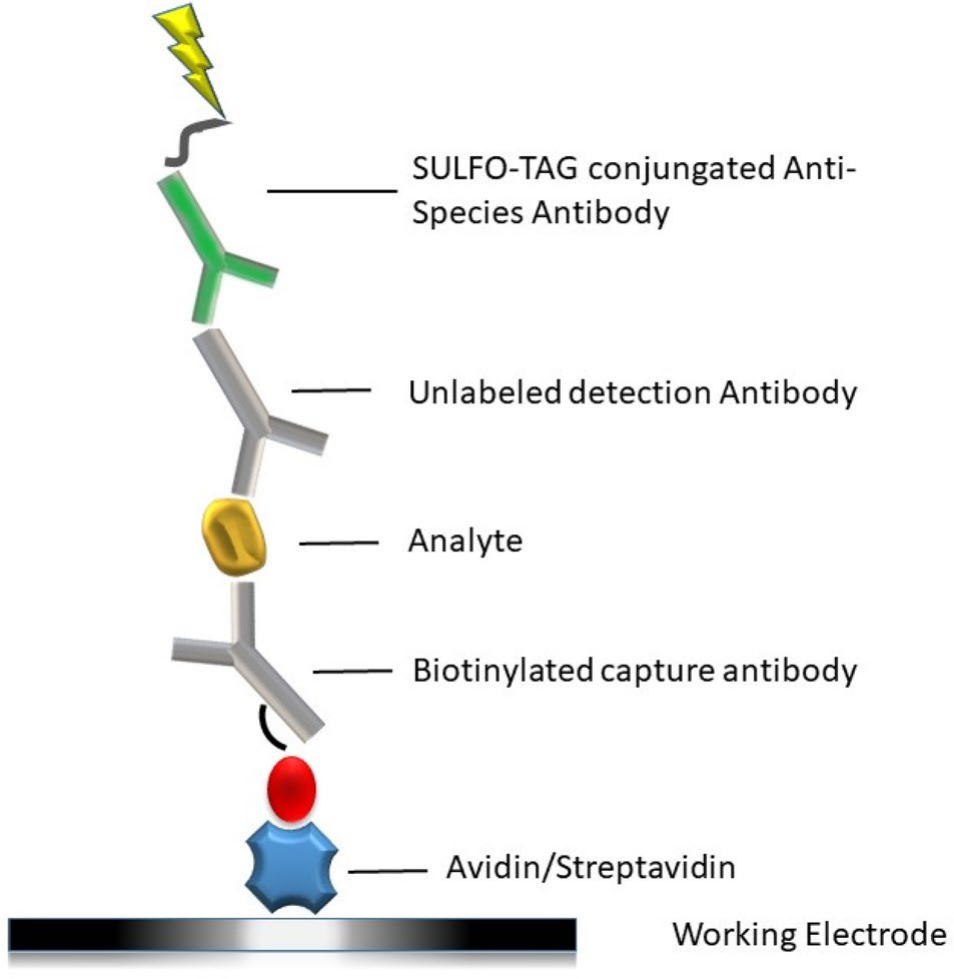
Structure of the immunoassay: a biotinylated capture antibody was coated on the streptavidin plate and after binding of the specific analyte, a primary detection antibody was added and for completion of the immunocomplex, a secondary detection antibody labeled with SULFO-TAG was used to produce the light signal.

The multiplex ECLIA assay was based on the U-Plex Development Pack from MSD (Catalog Number K15228N-2 Meso Scale Diagnostics, Rockville, Maryland) with the antibodies from the R&D DuoSet ELISA systems (Rat Total MMP-9, Catalog Number DY8174-05; Rat IL-17F, Catalog Number DY4437 and Rat ICAM-1/CD54 Catalog Number DY583). The assay used a sandwich immunoassay technique. A biotinylated capture antibody was added on the streptavidin-coated plate and after binding of the specific analyte, a primary detection antibody was added. To complete the immunocomplex subsequently, a secondary detection antibody labeled with SULFO-TAG (MSD^®^ SULFO-TAG Labeled Anti-Mouse Antibody, catalogue number: R32AC-5) was used to produce the light signal, as shown in Fig. 9.

Plates were coated with biotinylated capture antibodies and after incubation the plates were washed three times with 150 µl of 0.05% TWEEN^®^ 20 (Merck Life Science BV/SRL, catalogue number P2287) in Dulbecco’s phosphate buffered saline (DPBS, Gibco™) solution. Matrices were spiked with 7000, 1100 and 8500 pg/ml for the calibrators MMP-9, IL-17 and ICAM-1, respectively. Plates were incubated, washed three times, and coated with unlabeled detection antibody. After incubation, plates were washed three times and coated with SULFOTAG antibody, followed by another incubation and washing step. Then, the MSD Gold Read Buffer was added and plates were immediately read in the MESO QuickPlex SQ 120MM instrument. Standard conditions for incubations were 1 h at room temperature and shaking at 200 rpm.

### Analytical validation with the rat tear fluids

Analytical validation was performed with the rat tears diluted with assay diluent as matrix.

The second set of assays used tear fluid from each animal that was combined up to 50 µl and diluted 109 times with assay diluent to obtain enough sample for all the assays.

For precision, trueness, linearity, 2 plates from 2 operators in 2 different days were run. The assay format was made of 2 technical replicates for each animal.

Same conditions for testing robustness including three variables: incubation time (30/90 min), agitation (shaking/not shaking), and temperature of incubation (4 °C and 37 °C).

**Figure 10:**
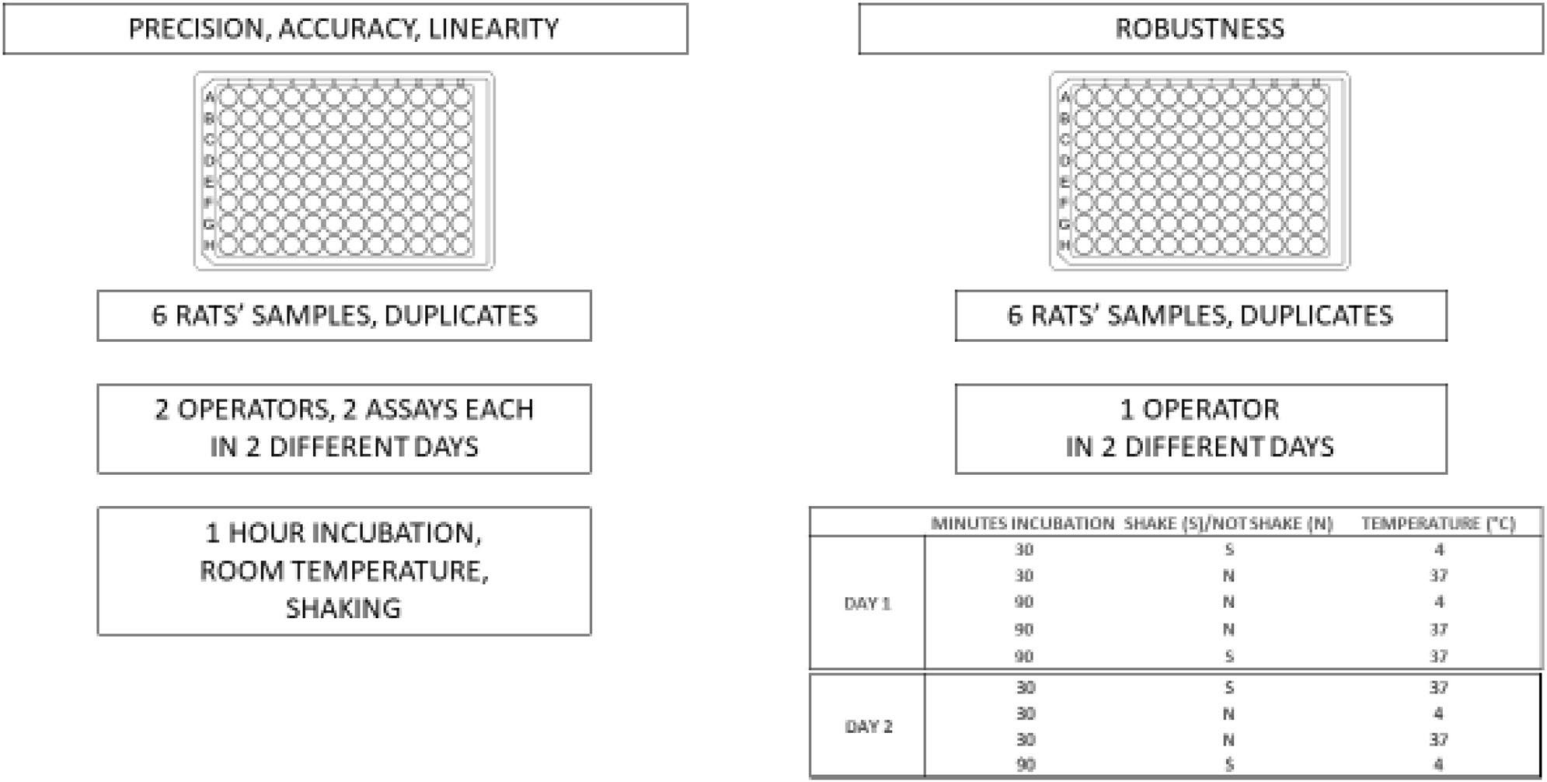
Experimental design of all assays runs. It is divided into two different main procedures for checking precision, accuracy and linearity parameters. They were used 6 samples from 6 rats and run in duplicates. Two independent operators run 2 assays each in 2 different days. The conditions were: 1 h of incubation at room temperature with the presence of shaking. Robustness assays were run by one single operator in two different days. In the figure they are listed all the combinations for testing incubation time, incubation temperature and the presence or absence of shaking.

The combinations tested on 2 different days by one operator are illustrated in Fig. 10.

### Statistical analysis

The analysis was firstly performed to evaluate the assay diluent matrix results.

To assess linearity, a linear model was fitted on the log transformed results versus the log transformed target (spiked) concentration for each analyte. The region target concentration over which the slope was closest to 1 and the intercept closest to 0 was searched for producing the graph of the residuals. Linearity was also assessed fitting a linear model on the log transformed results versus the log transformed target concentration for the results of each analyte using sample as random factor.

To assess precision and trueness, a linear model was fitted on the log transformed results by target concentration level and assay precision was estimated as well as the assay trueness. Overall precision was assessed fitting a linear model on the log transformed results using target concentration level as nominal fixed factor and sample as random factor.

To appraise robustness a linear model was fitted on the log transformed results with fixed effects being analytes, Log target concentration as continuous and time as categorical. The interactions evaluated were: analyte*Log target concentration, Log target concentration*time and time*analyte.

To assess the parameters on the tear fluid matrix data, a linear mixed model was fitted on the log transformed results for each analyte and target concentration level. The random effects included were day, operator, and plate. The repeatability variance was estimated by the residual variance. The intermediate precision of the assay was estimated by the sum of all variance components. The 95% upper confidence limits of the CVs were also given. The CV was computed as Eq. (2):

2 $$CV\left(\mathrm{\%}\right)=100\times \sqrt{\left({\mathrm{e}}^{{\widehat{\sigma }}^{2}}-1\right)}$$

With ${\widehat{\sigma }}^{2}$ the corresponding estimated variance of the log transformed data.

This model also allowed to estimate the mean log results per analyte and concentration level. This led to estimate the trueness of the assay by analyte and concentration level reporting them as recoveries together with their 95% confidence intervals. An evaluation of the overall assay precision on target concentration levels was performed with a linear mixed model fitted on the log transformed results and including the target concentration as fixed factor and day, operator, and plate as random factors.

For each analyte studied, linearity results were obtained by fitting a linear regression on the log transformed results of the experiments versus their target concentration after logarithmic transformation. Equation of the linear regression are given in the Results section, together with the 95% confidence interval of the intercept and slope as well as the determination coefficient (R^2^).

For each analyte, three robustness parameters have been studied over a range of target concentration: such as incubation time (30 min and 90 min), incubation temperature (4 °C and 37 °C) and agitation during incubation (shake and not shake). For each analyte a linear model was fitted to the logarithmic transformation of results (natural base) including the following fixed factors the main effects, such as time, temperature, agitation, target concentration after log transformation (natural base). The interactions taken into account are time vs temperature, time vs agitation, temperature vs agitation, time vs target concentration after log transformation (natural base), temperature vs target concentration after log transformation, agitation vs target concentration after log transformation.

## Conclusions

This is the first report of a validated ECLIA that allows measurements of three relevant DED biomarkers (MMP-9, IL-17 and ICAM-1) in rat tear fluids. Incubation time, temperature and agitation affected the robustness of the protocol. Precision was acceptable in a small range, while accuracy and linearity were acceptable for a broader range. This method can give an answer to the need of analytical methods for measuring the progress of DED in rat models.

## Supplementary Information

Supplementary Figure 1.

Supplementary Figure 2.

Supplementary Figure 3.

Supplementary Figure 4.

Supplementary Information.

Supplementary Table 1.

Supplementary Table 2.

Supplementary Table 3.

## Data Availability

The datasets generated during and/or analysed during the current study are available from the corresponding author on reasonable request.
