# Supplementary figures and images for: Development and validation of a multiplex electrochemiluminescence immunoassay to evaluate dry eye disease in rat tear fluids
Source: Sci Rep. 2023 Jul 27;13:12203. doi: 10.1038/s41598-023-39397-8 (PMC10374623; doi:10.1038/s41598-023-39397-8)

Supplementary Fig.1

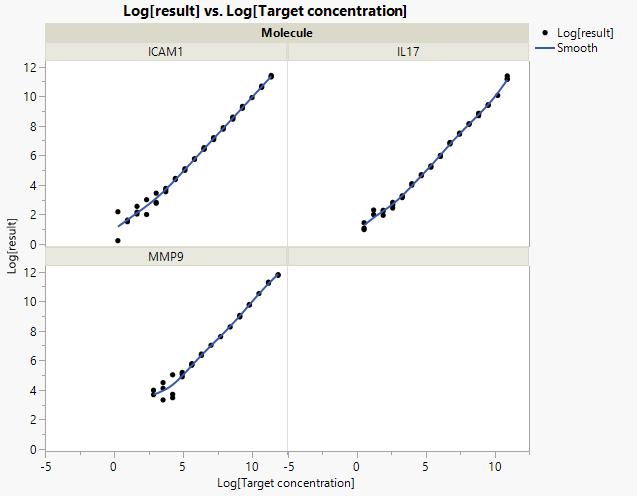

Supplement: Supplementary file 1 — Supplementary Figure 1. [file 41598_2023_39397_MOESM1_ESM.docx]

Supplementary Fig. 2


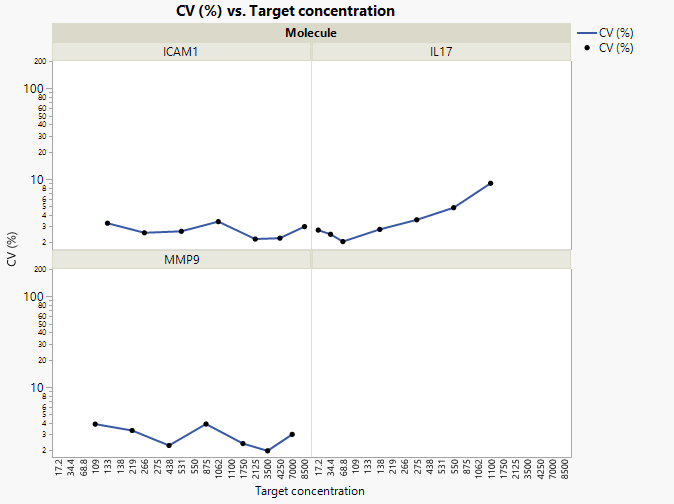

Supplement: Supplementary file 2 — Supplementary Figure 2. [file 41598_2023_39397_MOESM2_ESM.docx]

Supplementary Fig.3


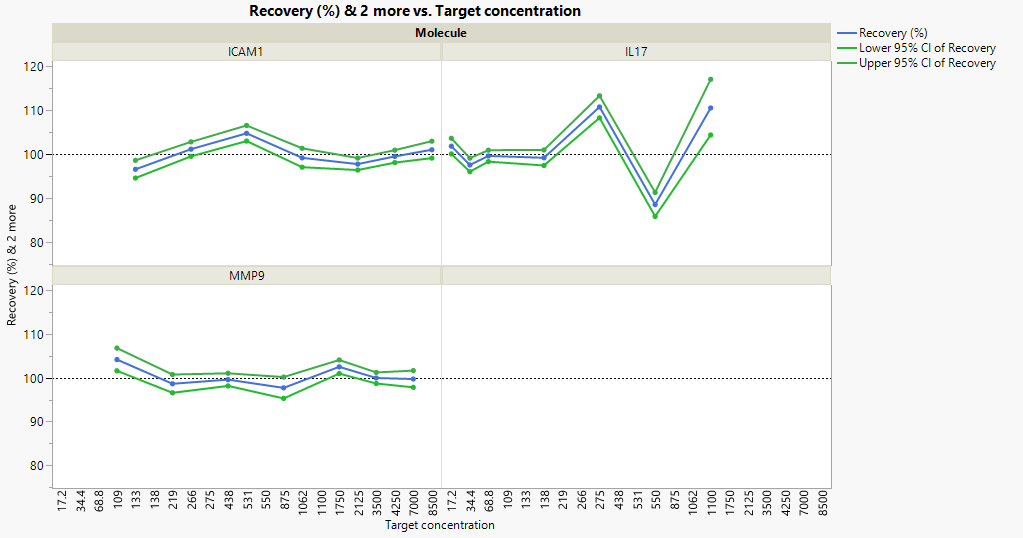

Supplement: Supplementary file 3 — Supplementary Figure 3. [file 41598_2023_39397_MOESM3_ESM.docx]
