# Supplementary material for: Development and validation of a multiplex electrochemiluminescence immunoassay to evaluate dry eye disease in rat tear fluids
Source: Sci Rep. 2023 Jul 27;13:12203. doi: 10.1038/s41598-023-39397-8 (PMC10374623; doi:10.1038/s41598-023-39397-8)
Supplement: Supplementary file 4 — Supplementary Figure 4. [file 41598_2023_39397_MOESM4_ESM.pptx]

## Slide 1
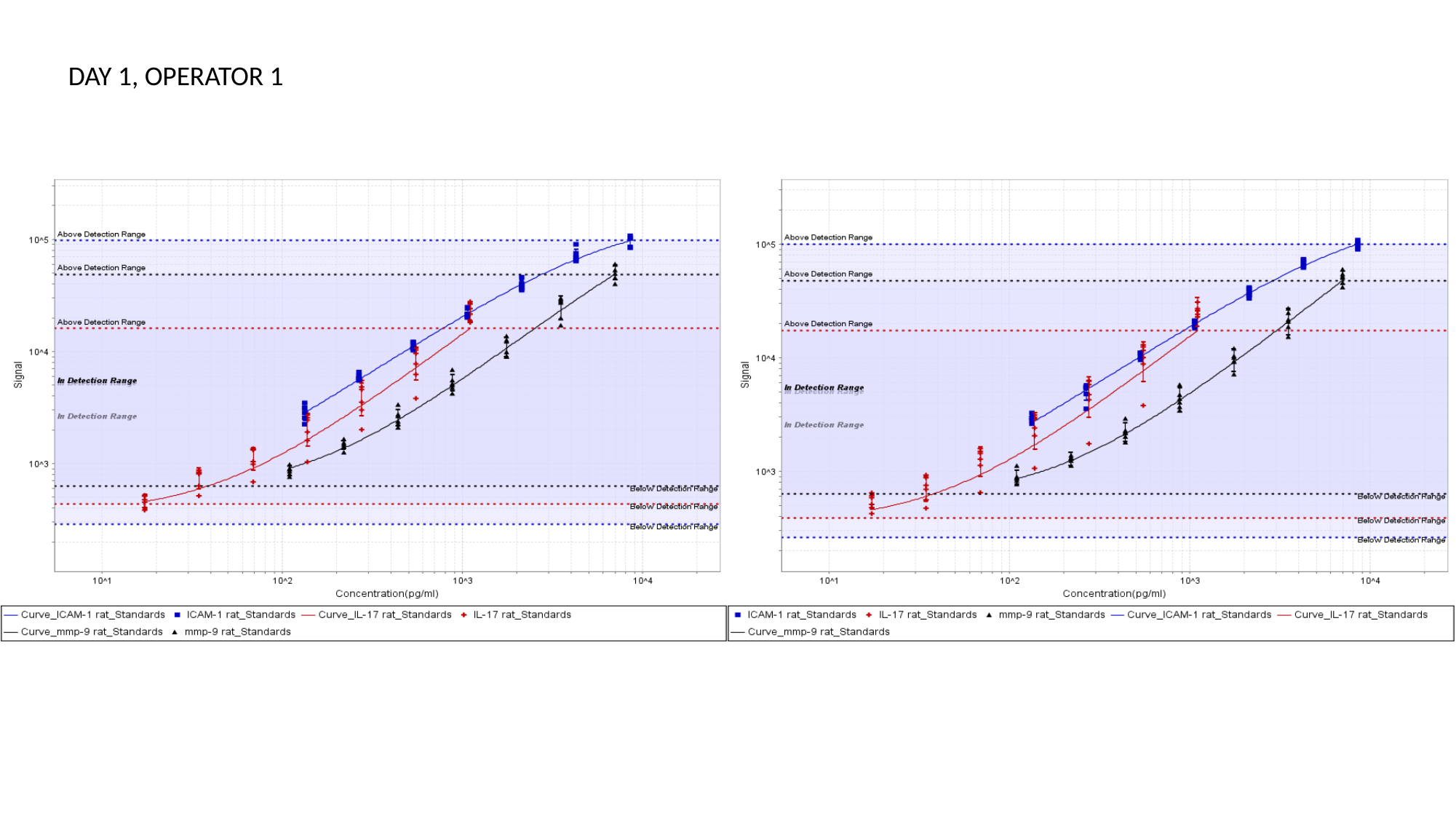

DAY 1, OPERATOR 1

## Slide 2
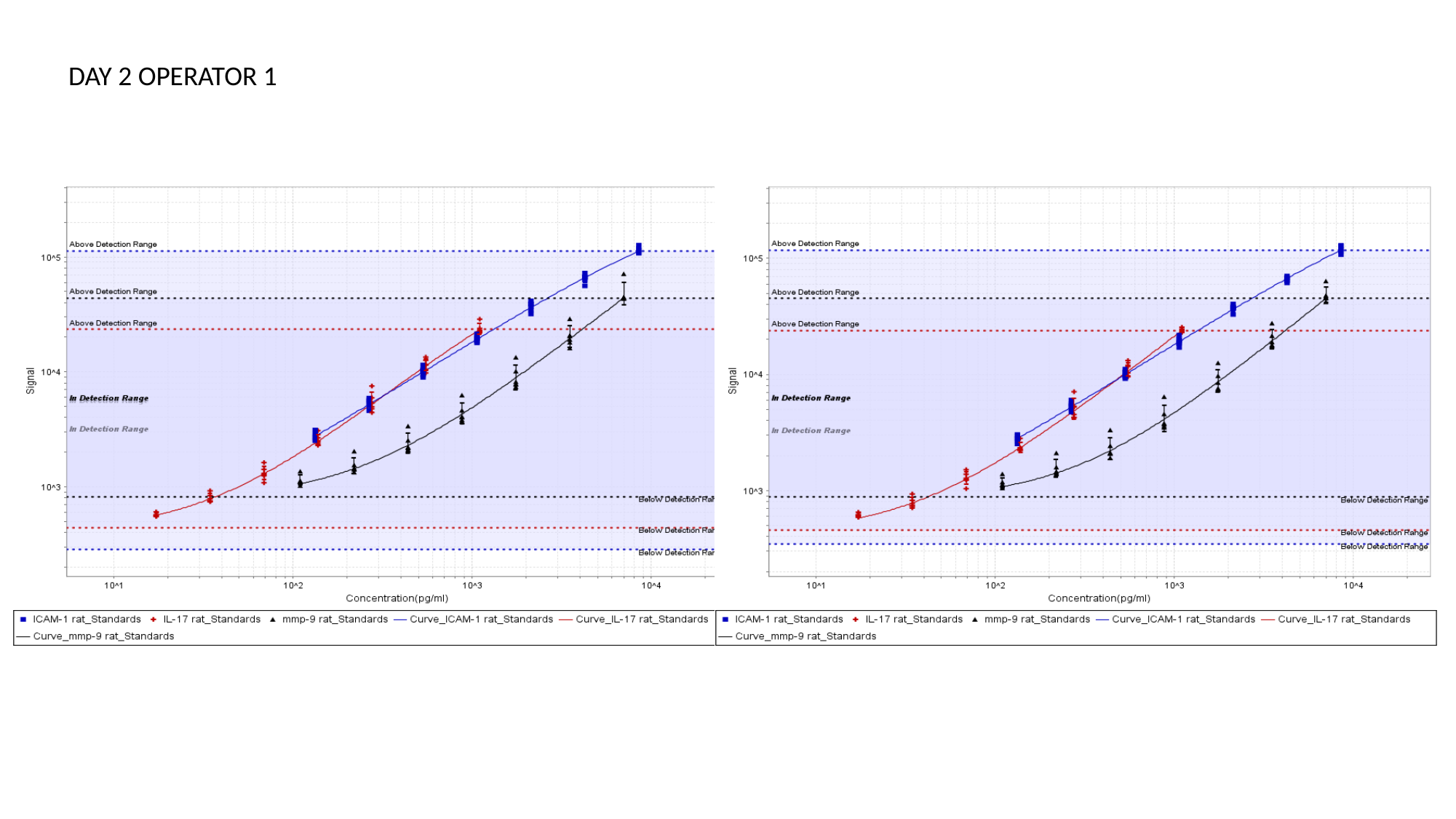

DAY 2 OPERATOR 1

## Slide 3
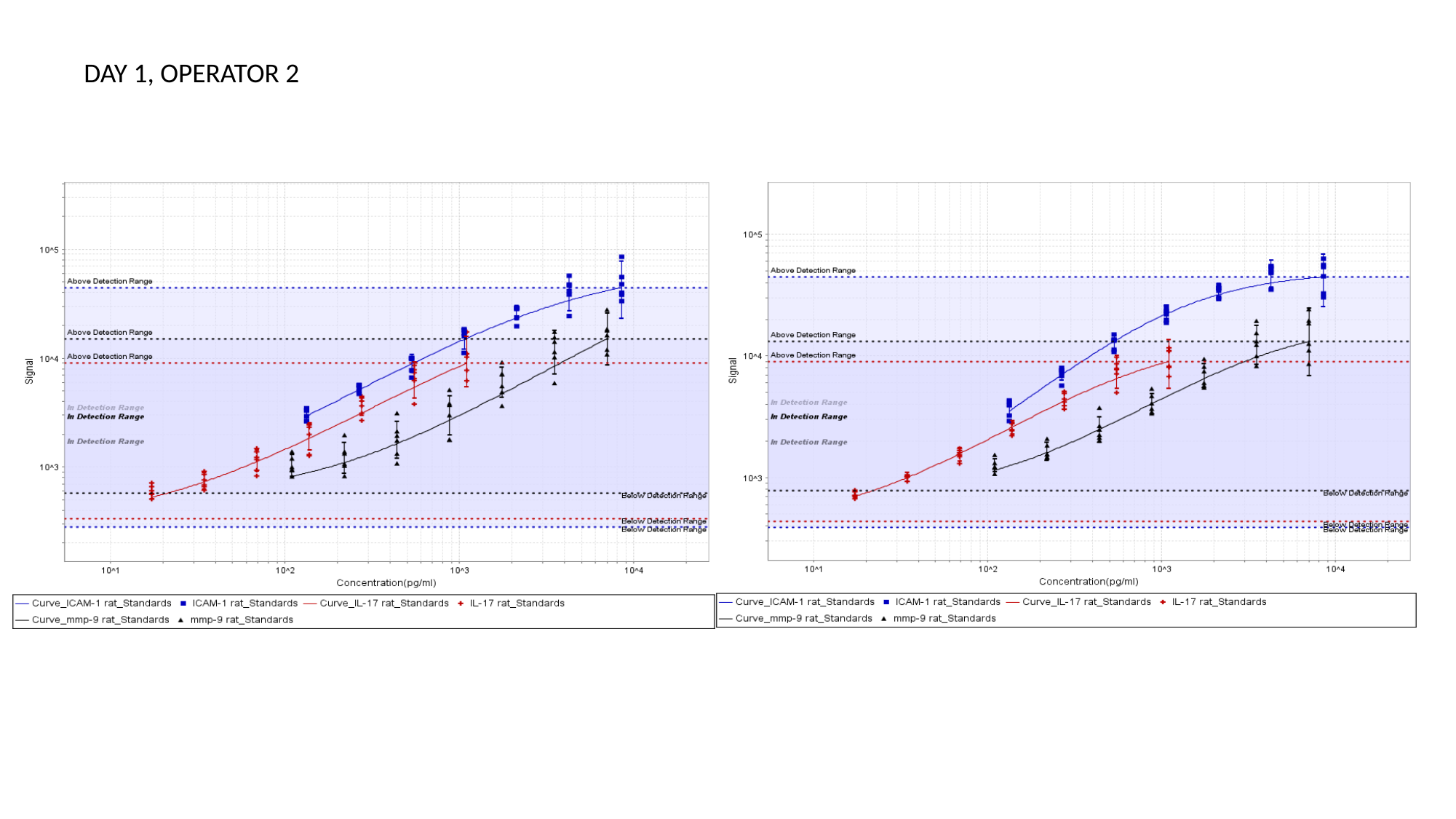

DAY 1, OPERATOR 2

## Slide 4
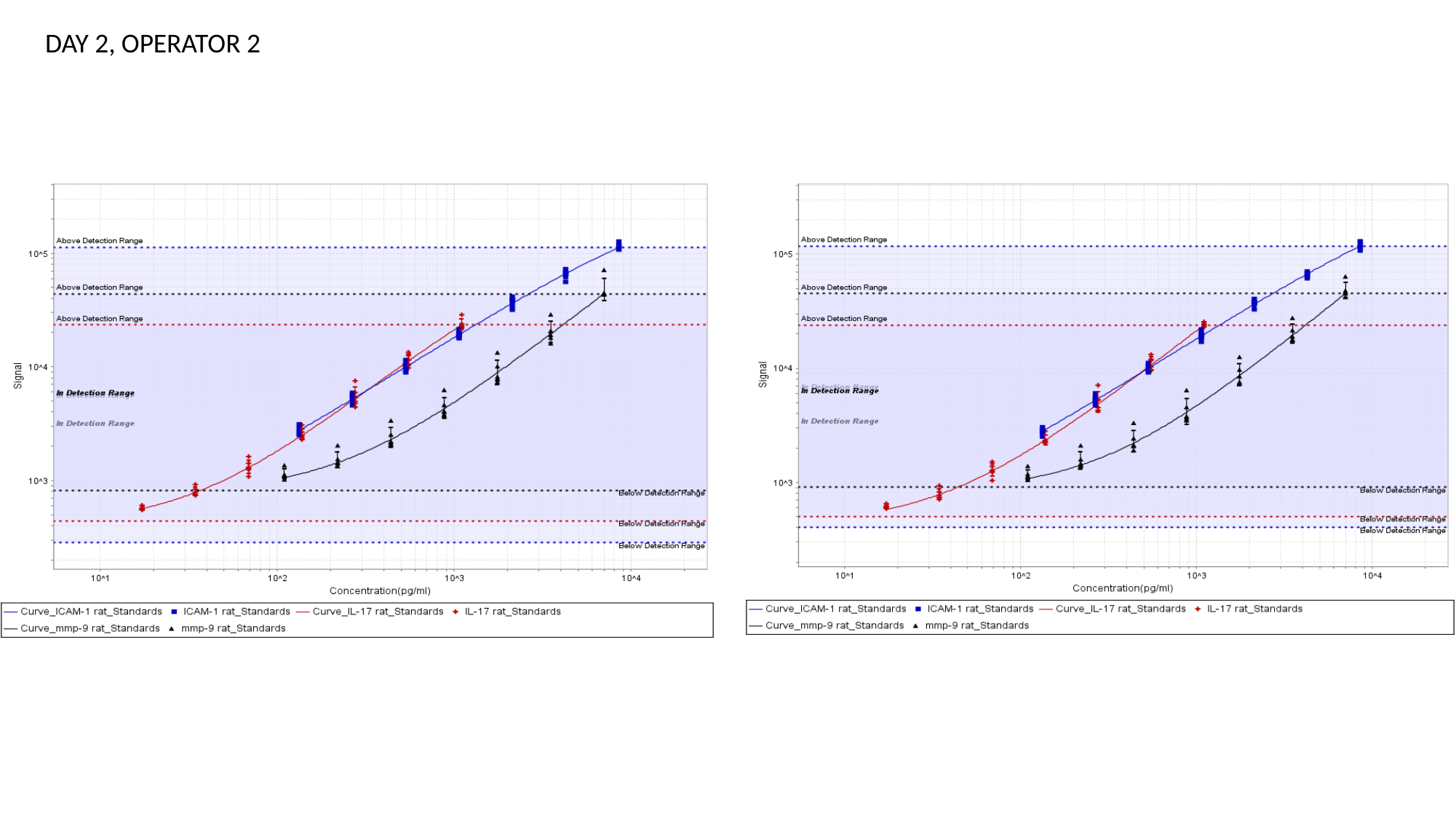

DAY 2, OPERATOR 2

## Slide 5
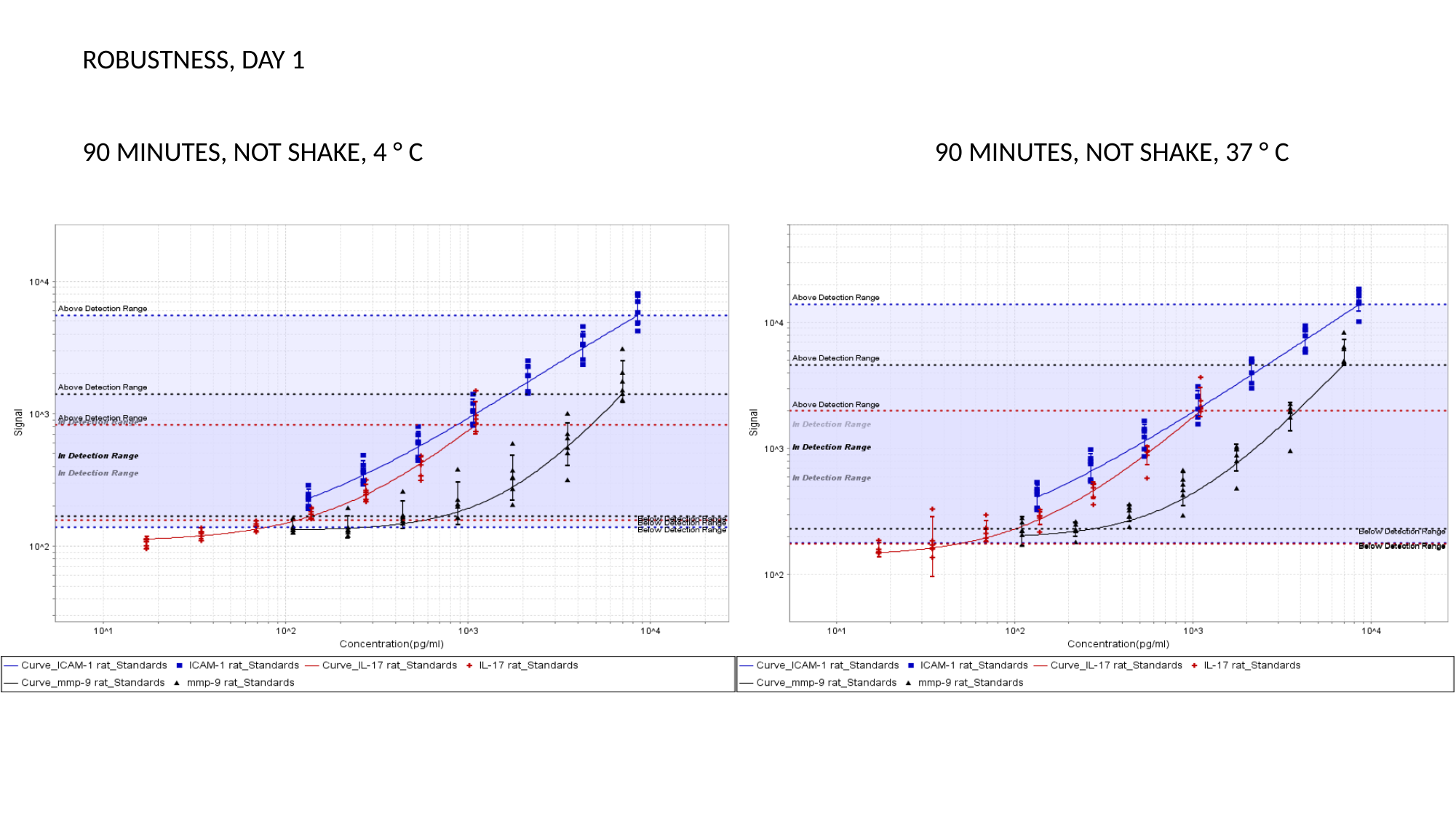

ROBUSTNESS, DAY 1
90 MINUTES, NOT SHAKE, 4 ° C
90 MINUTES, NOT SHAKE, 37 ° C

## Slide 6
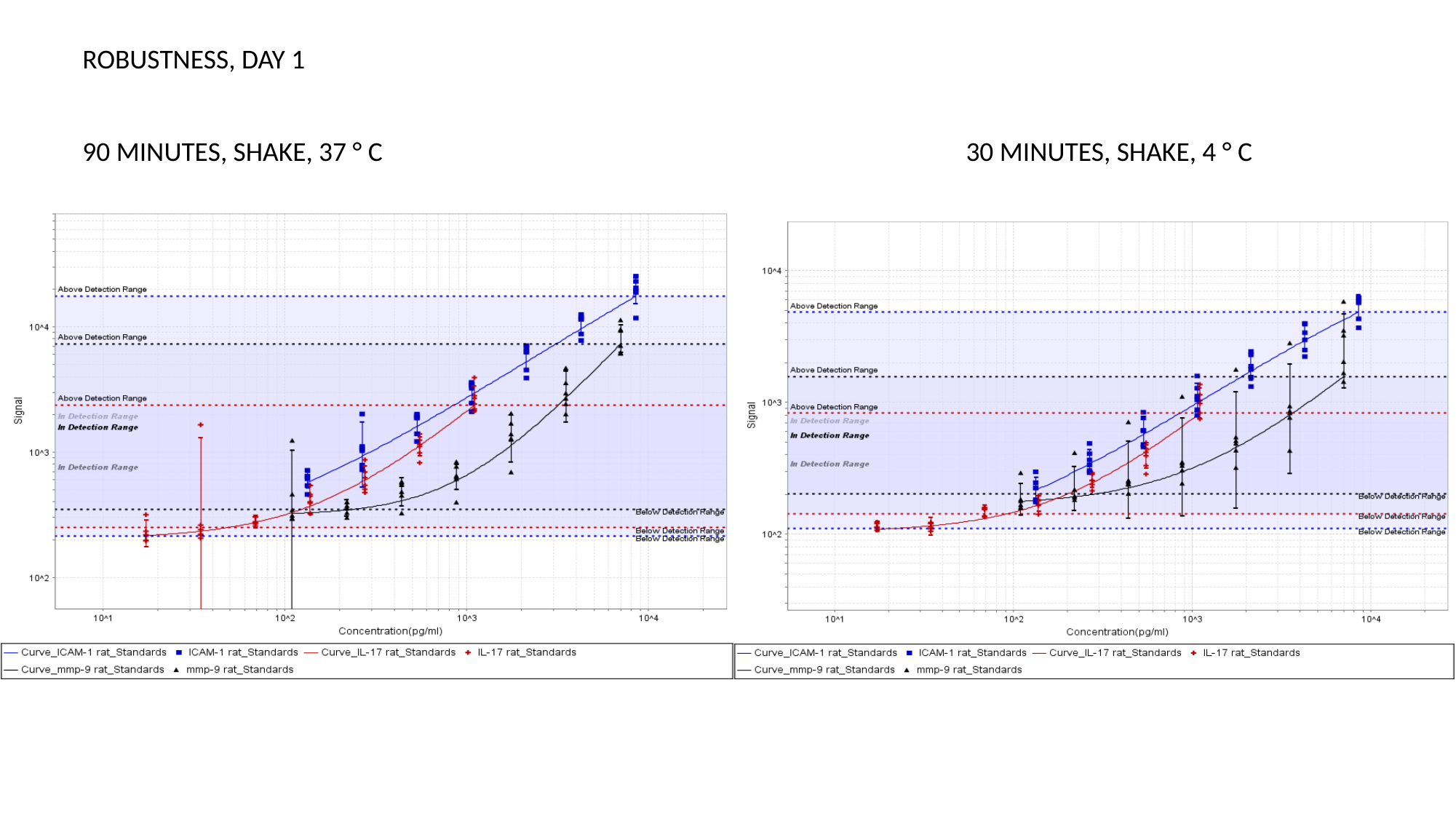

ROBUSTNESS, DAY 1
90 MINUTES, SHAKE, 37 ° C
30 MINUTES, SHAKE, 4 ° C

## Slide 7
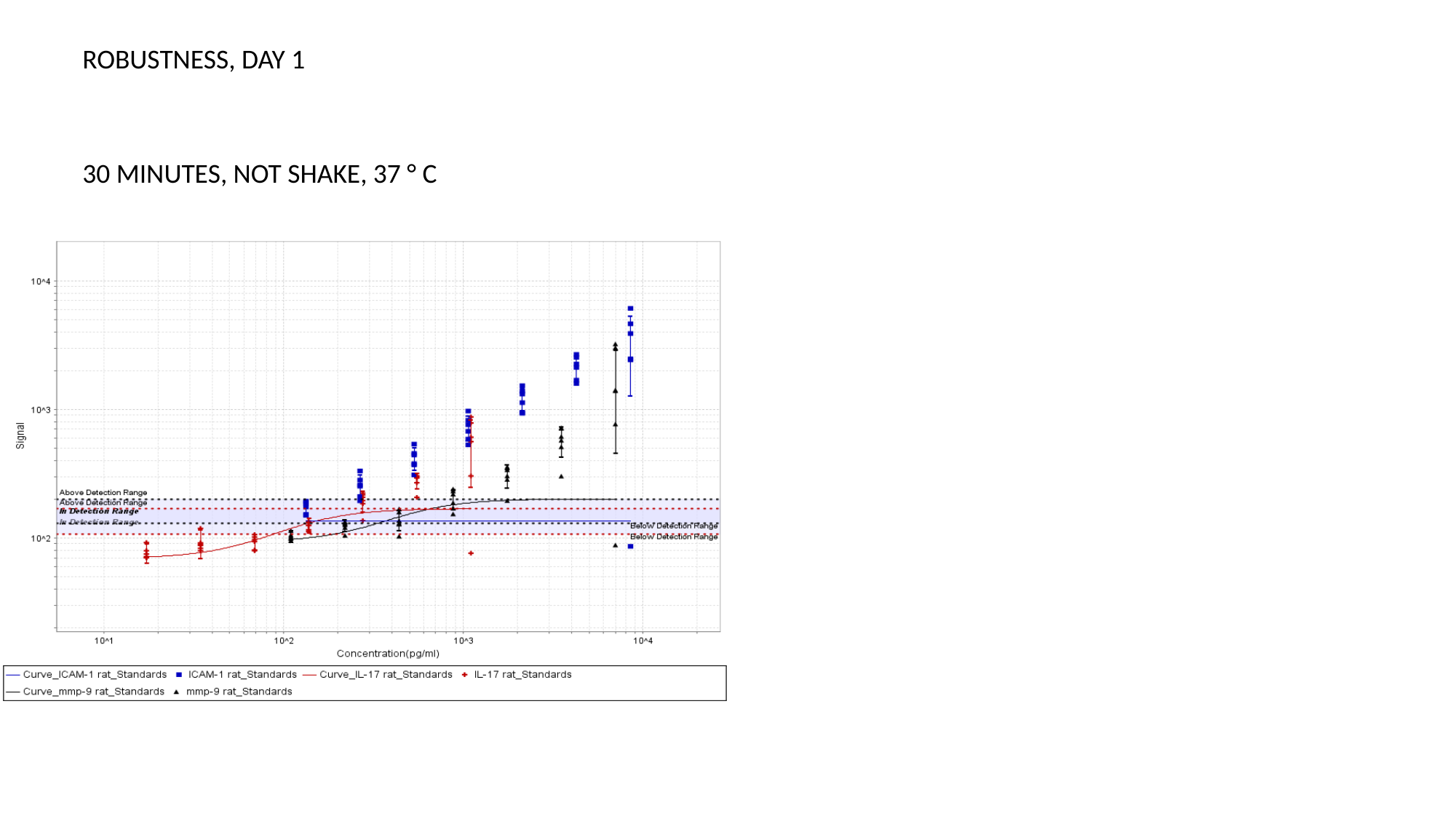

ROBUSTNESS, DAY 1
30 MINUTES, NOT SHAKE, 37 ° C

## Slide 8
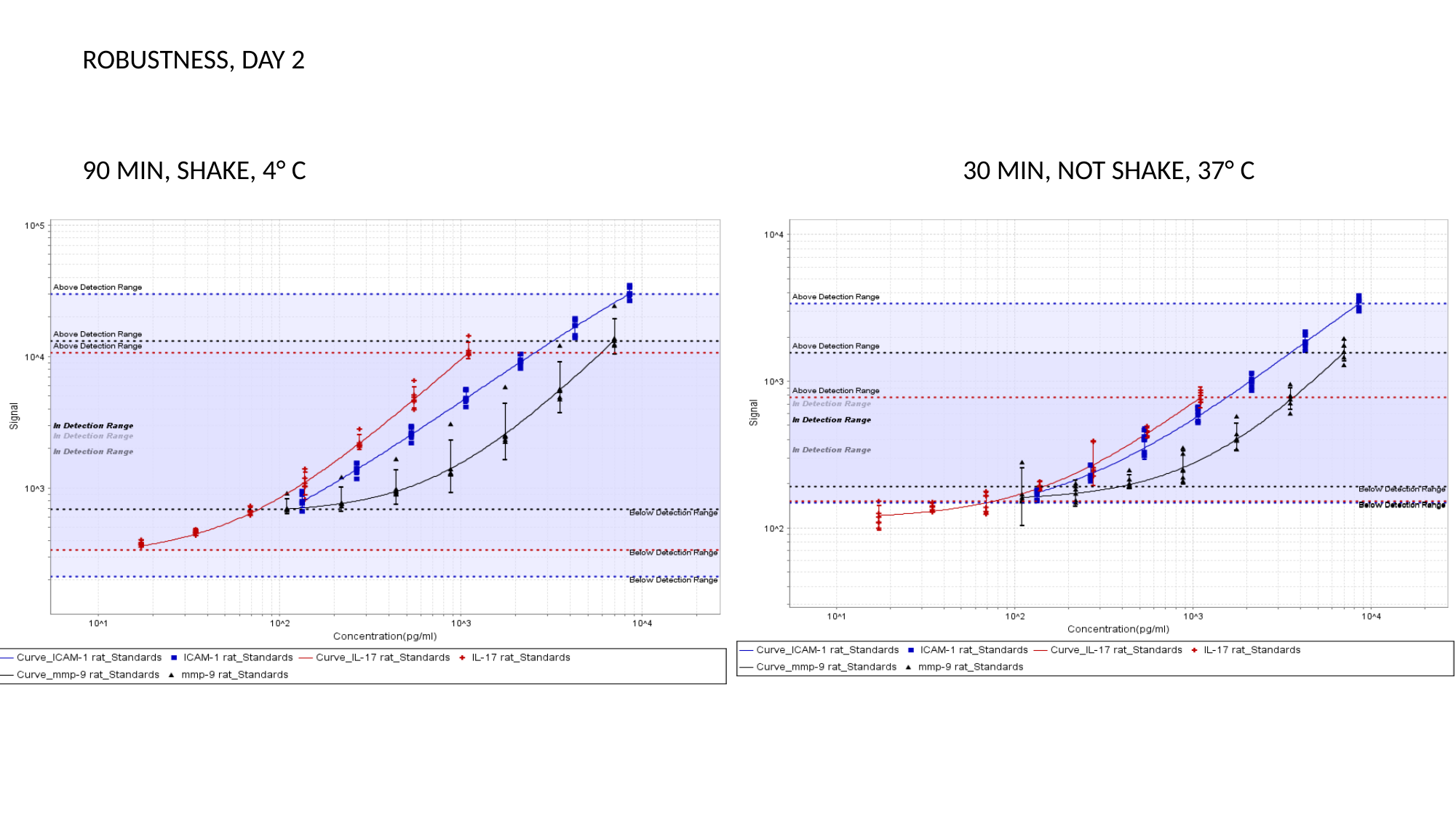

ROBUSTNESS, DAY 2
90 MIN, SHAKE, 4° C
30 MIN, NOT SHAKE, 37° C

## Slide 9
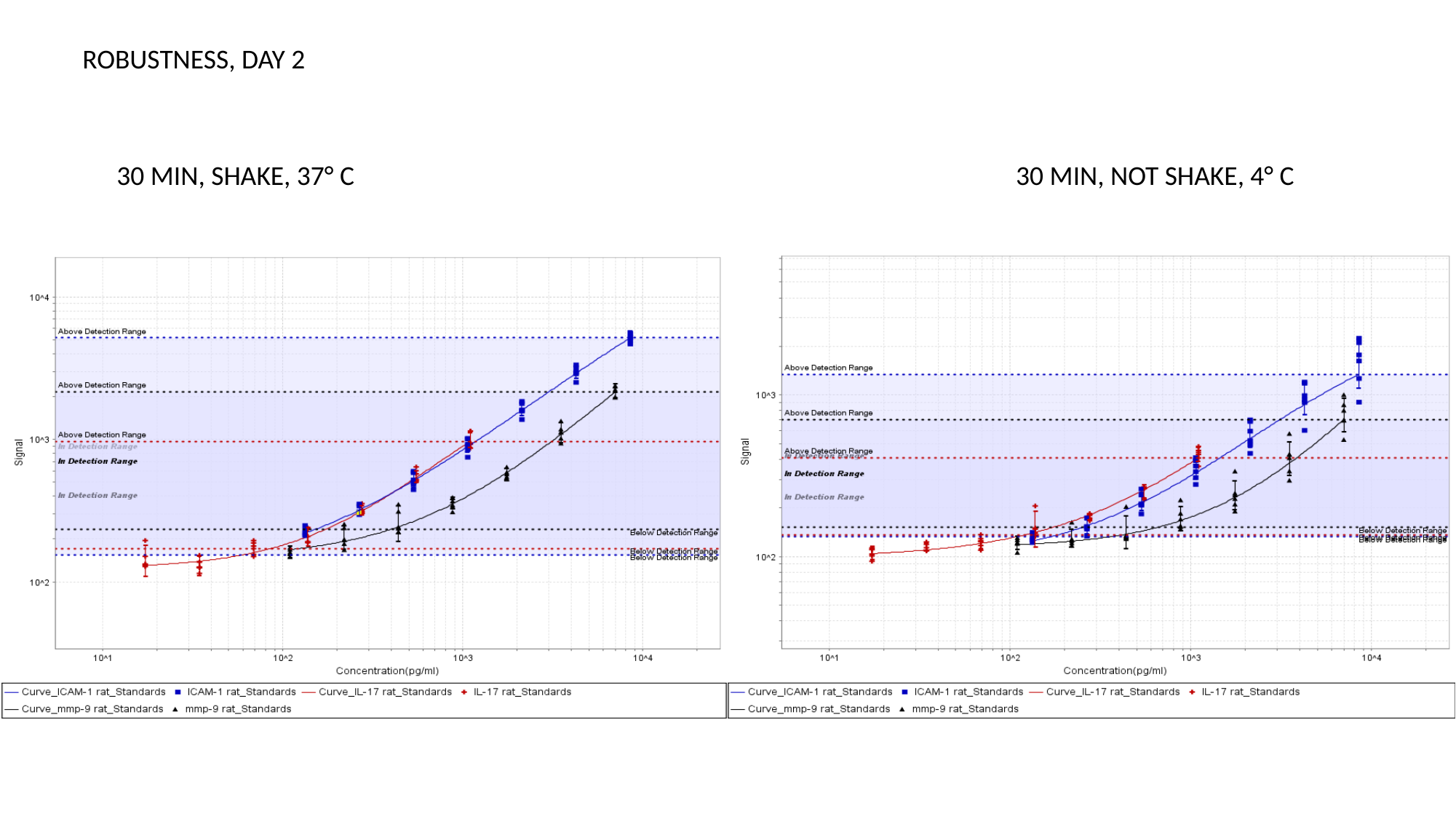

ROBUSTNESS, DAY 2
30 MIN, SHAKE, 37° C
30 MIN, NOT SHAKE, 4° C
