# Supplementary material for: Development and validation of a multiplex electrochemiluminescence immunoassay to evaluate dry eye disease in rat tear fluids
Source: Sci Rep. 2023 Jul 27;13:12203. doi: 10.1038/s41598-023-39397-8 (PMC10374623; doi:10.1038/s41598-023-39397-8)
Supplement: Supplementary file 5 — Supplementary Information. [file 41598_2023_39397_MOESM5_ESM.docx]

Preliminary data ECLIA using assay diluent matrix
Dilution linearity

Dilution linearity demonstrates accuracy for samples with concentrations above the upper limit of quantification (ULOQ) after dilution into the assay range. Supplementary Figure 1 plots the Log of the result versus the Log of the target concentration spiked on the samples. The figure shows that linearity is in general acceptable, but plainly satisfied at higher concentrations. The results from the statistical analysis are illustrated in Supplementary Table 1. The intercept can be used as an indicator of bias, which is the persistent difference between the value of the test result and the reference quantity. Therefore, between the two factors there is a proportional relationship. An intercept equal to zero represents a bias-free situation. R² values of the intercept for every analyte display the quality of the fit and thus the linearity. R² values close to 1 confirm the well pronounced linearity of the method.

Precision

Precision is usually expressed as coefficient of variance (CV). Values in this analysis never exceed 15% CV and all the results are within the CV range indicated in the validation requirements for precision. Data are shown in Supplementary Fig.2.

Trueness

Trueness, also mentioned as accuracy, can be assessed through recovery which is the difference between the measured concentration of the spiked sample and the one in the neat sample, divided by the theoretical spiked concentration, commonly expressed in %. Analysis shows that all the values fall within the acceptance range of 80-120% (Supplementary Fig. 3).

Robustness

Robustness data were obtained using spiked assay buffer matrix and incubation times of 30 and 90 minutes. Supplementary Table 2 shows the effects analyzed and based on the p-value, indicated as Prob>F. There is a considerable effect of the target concentration on the results measured, which was expected. Moreover, there is no statistically substantial effect of the time, whatever the analyte and the level of the target concentration.

Methods

Samples

Preliminary data were obtained using the spiked assay diluent as matrix. After optimization with the use of this matrix, validation of the multiplex ECLIA was performed on the rat tear fluids.

Development and optimization of the multiplex ECLIA design with assay diluent matrix

The entire development of the multiplex ECLIA followed numerous steps. First, the performance of a single analyte ECLIA was tested to examine the optimal concentrations of the reagents in the immunoassay.
Based on the signal to background ratios of single ECLIA assays the optimal reagent concentration was chosen for each assay and for each analyte. This data is summarized in Supplementary Table 3. These three single ECLIAs were performed using twelve samples, whereby each column was a sample and the eighth well for each replicate contained the blank.
All data were analyzed using MSD Workbench 4.0 software. The software fits the standard curves using a 4-parameter logistic fit with 1/y^2^ weighting. The 4-PL equation ( 2 ) is:

$$y=b_{1}+\frac{b_{2}-b_{1}}{1+\left( x/b_{3} \right)^{b_{4}}}$$

( 2 )

Where y = signal, x = concentration, b2 = estimated response at infinite concentration, b1 = estimated response at zero concentration, b3 = mid-range concentration, b4 = slope factor

One multiplex ECLIA was then performed to find the concentration with the highest signal to background ratio of every step and to check for the absence of cross reactivity.

Cross reactivity was evaluated with the use of the following equation (3):

$$\% Cross reactivity of analyte A with antibody B = (Signal B - Bkg B) / (Signal A - Bkg A) x 100$$

( 3)

Where signal= value estimated by the equipment, Bkg= background value.

No cross reactivity was found and the optimal concentration for every step was 0,5 µg/ml for capture, detection, and secondary detection antibodies (data not shown).

The biotin ECLIAs were further performed to test the validation parameters, using the assay diluent as matrix and the experimental design based on Andreasson et al. 2015 [20]. Precision was evaluated with 12 technical replicates and seven 2-fold dilutions of the calibrator. Robustness was evaluated with equivalent settings, testing different incubation times of 30 and 90 minutes for each step. Dilution linearity was evaluated with 3 technical replicates in which the calibrators were spiked at the maximum concentration allowed: 70000, 27500 and 85000 pg/ml for MMP-9, IL-17 and ICAM-1 respectively for 22 two-fold dilutions. Sixteen wells were run as blank.

FIGURE LEGENDS

Supplementary Figure 1 Linearity: linear model fitted on the log transformed results versus the log transformed target concentration for each analyte. Regions where concentration over which the slope is closest to 1 and intercept closest to 0 are targeted. N= 12 samples.

Supplementary Figure 2: Precision: linear model fitted on the log transformed results by target concentration level expressed in pg/ml. N= 12 samples

Supplementary Figure 3: Trueness: Recovery% and upper and lower confidence intervals vs. target concentration expressed in pg/ml. N= 12 samples
