# Supplementary material for: Development and validation of a multiplex electrochemiluminescence immunoassay to evaluate dry eye disease in rat tear fluids
Source: Sci Rep. 2023 Jul 27;13:12203. doi: 10.1038/s41598-023-39397-8 (PMC10374623; doi:10.1038/s41598-023-39397-8)
Supplement: Supplementary file 6 — Supplementary Table 1. [file 41598_2023_39397_MOESM6_ESM.docx]

| **Analyte** | **Term** | **Estimate** | **Lower 95% CI** | **Upper 95% CI** | **R²** | **Concentration Range** |
| --- | --- | --- | --- | --- | --- | --- |
| ICAM-1 | Intercept | -0.0124 | -0.0505 | 0.0257 | 0.9995 | [133-8500] |
| ICAM-1 | Log[Target concentration] | 1.0017 | 0.9965 | 1.0069 |  |  |
| IL-17 | Intercept | -0.0314 | -0.0982 | 0.0355 | 0.9965 | [17.2-1100] |
| IL-17 | Log[Target concentration] | 1.0081 | 0.9951 | 1.0212 |  |  |
| MMP-9 | Intercept | 0.0291 | -0.0117 | 0.07 | 0.9995 | [109-7000] |
| MMP-9 | Log[Target concentration] | 0.9961 | 0.9902 | 1.02 |  |  |

Supplementary Table 1: Linearity: a linear model is fitted on the log transformed results versus the log transformed target concentration results for each analyte using sample as random factor. N= 12 samples. Concentration range is expressed in pg/ml.
