# Supplementary material for: Development and validation of a multiplex electrochemiluminescence immunoassay to evaluate dry eye disease in rat tear fluids
Source: Sci Rep. 2023 Jul 27;13:12203. doi: 10.1038/s41598-023-39397-8 (PMC10374623; doi:10.1038/s41598-023-39397-8)
Supplement: Supplementary file 7 — Supplementary Table 2. [file 41598_2023_39397_MOESM7_ESM.docx]

| **Effect Tests** |  |  |  |  |  |  |  |
| --- | --- | --- | --- | --- | --- | --- | --- |
| Source | | | Nparm | DF | Sum of Squares | F Ratio | Prob > F |
|  |  |  |  |  |  |  |  |
| Log[Target concentration] | | | 1 | 1 | 957.61197 | 123464.9 | <.0001* |
| analyte |  |  | 2 | 2 | 2.2174 | 2.2174 | 0.11 |
| Log[Target concentration]*analyte | | | 2 | 2 | 1.8278 | 1.8278 | 0.1619 |
| time |  |  | 1 | 1 | 0.2317 | 0.2317 | 0.6305 |
| time*analyte | |  | 2 | 2 | 0.4766 | 0.4766 | 0.6212 |
| Log[Target concentration]*time | | | 1 | 1 | 1.0893 | 1.0893 | 0.2971 |

Supplementary Table 2: Results of linear model fitted on the log transformed results with fixed effects including “analytes”, “Log target concentration” and “time” and their interactions. The column Prob>F shows the p-value of the corresponding effect. The columns “Nparm” and “DF” show the number of parameters and degrees of freedom, respectively, of the analysis. N= 12 samples.
