# Supplementary material for: Development and validation of a multiplex electrochemiluminescence immunoassay to evaluate dry eye disease in rat tear fluids
Source: Sci Rep. 2023 Jul 27;13:12203. doi: 10.1038/s41598-023-39397-8 (PMC10374623; doi:10.1038/s41598-023-39397-8)
Supplement: Supplementary file 8 — Supplementary Table 3. [file 41598_2023_39397_MOESM8_ESM.docx]

|  | MMP-9 | IL-17 | ICAM-1 |
| --- | --- | --- | --- |
| BIOTINYLATED CAPTURE ANTIBODY (µg/ml) | 1 – 0.5 | 1 – 0.5 | 1 – 0.5 |
| CALIBRATOR (pg/ml) | 7000 | 1100 | 8500 |
| UNLABELED DETECTION ANTIBODY (µg/ml) | 10 - 1 – 0.5 | 1 – 0.5 – 0.25 | 1 – 0.5 – 0.25 |
| SECONDARY DETECTION ANTIBODY (µg/ml) | 1 – 0.5 | 1 – 0.5 | 1 – 0.5 |

Supplementary Table 3: Optimal reagent concentrations tested for each assay and analyte. Calibrators were run in seven 2-fold dilutions starting from the value indicated. The second detection antibody was always labelled with SULFO-TAG. N= 12 samples.
